# Enhanced cycle life of starter lighting ignition (SLI) type lead–acid batteries with electrolyte modified by ionic liquid†

**DOI:** 10.1039/d3ra04386j

**Published:** 2023-08-07

**Authors:** Paweł Kędzior, Waldemar Rzeszutek, Jarosław Wojciechowski, Andrzej Skrzypczak, Grzegorz Lota

**Affiliations:** a PPUH Autopart Jacek Bąk sp. z o.o. Kwiatkowskiego 2A Mielec 39-300 Poland; b Institute of Chemistry and Technical Electrochemistry, Poznan University of Technology Berdychowo 4 Poznań 60-965 Poland grzegorz.lota@put.poznan.pl jaroslaw.g.wojciechowski@put.poznan.pl; c Łukasiewicz Research Network – Institute of Non-Ferrous Metals Division in Poznan, Central Laboratory of Batteries and Cells Forteczna 12 61-362 Poznan Poland

## Abstract

The aim of the presented work was to improve the lifetime of lead–acid SLI (starting, lighting and ignition) batteries through electrolyte modification with ionic liquids. The conducted research included the synthesis and determination of the influence of di(hexadecyldimethylammonium) and di(octadecyldimethylammonium) sulphates on the basic parameters (capacity, cranking performance) of the starter battery as well as parameters affecting its lifetime (dynamic charge acceptance, corrosion, water consumption). It has been shown that the addition of these compounds increases corrosion resistance and reduces water consumption, resulting in an increase in cyclic durability by up to 36%. The improvement is associated with the absorption of ionic liquid molecules into the mass of lead(ii) sulphate, which was confirmed by physicochemical and electrochemical studies.

## Abstract 2

The addition of ionic liquids to the sulfuric acid electrolyte solution in a lead–acid battery inhibits corrosion of current collectors and sulfation of the negative electrode. This increases the service life of the entire device.

## Graphical abstract

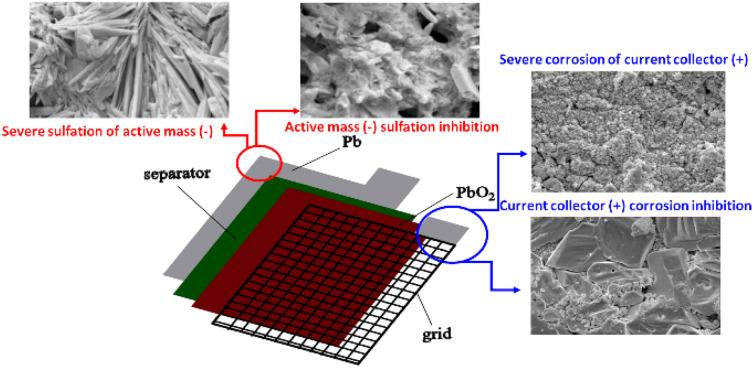

## 1 Introduction

Chemical power sources play a significant role in everyday life and various industrial sectors. Nowadays, it is increasingly difficult to find a device that does not require power or support from an electrical energy source. This translates into higher requirements and technological advancements in the construction of cells and batteries (accumulators).^1^ Despite the dynamic development of lithium-ion battery technology in recent years, lead–acid batteries are still dominant in the market due to their low raw material costs, low energy consumption during production, high recycling efficiency, and reliable system operation.^2–4^ Lead–acid batteries are mostly utilized in the automotive industry in the form of starting, lighting, and ignition (SLI) batteries types.^5^ However, with the current rapid development of fully electric vehicles, they can also serve as auxiliary batteries, providing safety features.^6^ This requires specially designed high-energy devices with high cyclic durability.^7–9^

A significant part of the current research on lead–acid batteries focuses on various additives for the negative and positive active electrode masses, as well as for the electrolyte. In the case of the negative electrode, commonly used additives include lignosulfonates, barium sulphate, and carbon in various allotropic forms.^10^ Carbon additives are currently the most intensively researched.^11^ In the case of the electrolyte, phosphoric acid and sodium sulphate are often used.^12,13^ There are also literature reports on the effects of surfactants and ionic liquids.^14–26^ The electrolyte is one of the active materials of the lead–acid battery and comes into contact with all elements of the system. Modifying its composition can significantly affect the utilization of the active materials in both the negative and positive electrodes, as well as reduce the internal resistance of the entire device. Additives used in the electrolyte should meet a number of requirements due to the highly severe environment. First and foremost, they must be electrochemically, chemically, and thermally stable in concentrated sulfuric acid (vi) under the conditions of production and operation of the lead–acid battery. Ionic liquids, which are defined as salts with melting temperatures below 373 K, fulfill these requirements.^27^ Below the melting point, these compounds are a liquids consisting only of ions. Due to their low vapor pressure and high thermal and chemical stability, ionic liquids are considered as environmentally friendly. This is a very wide group of compounds that has found application in most branches of electrochemistry. A particularly large share is visible in chemical power sources.^28,29^ In the case of lead–acid batteries, Rezaei *et al.* found that different ammonium bisulphates affects the stability of the electrolyte and inhibits the phenomenon of electrochemical corrosion of current collectors.^16–18^ Additionally, it has been shown that the observed effects depend on the concentration of the ionic liquid and the number of branches and length of alkyl substituents.^19^ However, in some cases, the authors identified negative effects of ionic liquid additives, such as the formation of larger crystals of lead(ii) sulphate and an increase in the rate of corrosion phenomenon.^20^ A significant effect of the addition of ammonium ionic liquids to the electrolyte on the hydrogen evolution potential was also conformed.^21^ Deyab *et al.* obtained a synergistic effect of using the phosphate anion and the imidazolium cation. An increase in battery capacity was observed together with the simultaneous inhibition of corrosion of current collectors and the inhibition of hydrogen gas evolution.^22^ Studies on the effect of the addition of ionic liquid with polydiallyldimethylammonium cation and bisulphate anion also showed an increase in capacity and a decrease in internal resistance, as well as a significant decrease in the corrosion rate of the positive electrode current collector and an improvement in the electrochemical stability of the electrolyte. Similar effects were also observed for diallyldimethylammonium, hexyldimethylammonium, and hexyltrimethylammonium sulphates, with the greatest effect observed for the polymeric ionic liquid.^23^ Kopczyński *et al.* estimated the dependence of electrolyte stability and corrosion rate of battery current collectors on the length of the alkyl chain in alkyl trimethylammonium bisulphates and sulphates.^24^ However, it was noted that the practical application of the investigated ionic liquids in the presented concentrations is not possible due to the intense foaming effect.^25^ Additionally, dimethylalkylammonium sulphate additives with alkyl substituents ranging from 4 to 16 carbon atoms in the chain were also studied. Based on this, it was found that the presence of dimethylalkylammonium ionic liquid in the positive electrode active mass leads to an increase in capacity during cyclic operation, a decrease in internal resistance, inhibition of corrosion processes, and an increase in self-discharge of the battery.^26^

The aim of the work is to assess the effect of the addition of ammonium ionic liquids to the electrolyte solution on the cyclic durability of SLI type lead–acid batteries. The possibility of practical application of ionic liquids in the production of batteries was taken into account. The lifetime of the battery system is significantly affected by the corrosion of the positive electrode current collectors, charge acceptance and water consumption.^9^ The influence of ionic liquids on these parameters was estimated using DC and AC electrochemical techniques. In addition, the surfaces of the individual elements of the battery were also characterized by physicochemical tests. The presented results are a direct response to the main challenges faced by SLI systems.

## 2 Experimental

### 2.1. Ionic liquids selection, synthesis and physicochemical analysis

Two different compounds with the following formulas were obtained: 2[(CH_3_)_2_HNC_16_H_33_]^+^ SO_4_^2−^ (di(hexadecyldimethylammonium) sulphate) and 2[(CH_3_)_2_HNC_18_H_37_]^+^ SO_4_^2−^ (di(octadecyldimethylammonium) sulphate). Ionic liquids selection, reagents for synthesis, synthesis and physicochemical analysis (NMR, elemental and thermal) procedures are given in detail in ESI.†

### 2.2. Weight loss corrosion test of current collectors

The cell for corrosion testing (weight loss method) consisted of two electrodes connected to the negative terminals and a Pb–Ca–Sn–Al alloy polarized anodically (positive terminal). The negative electrodes were placed on both sides of the positive electrode. They are Pb–Ca–Al and Pb–Ca–Sn–Al alloys, respectively. All of the electrodes were a grids produced by Autopart company using the expanded metal technology. The test cell was filled with an electrolyte with the addition of ionic liquid in concentrations of 0.1, 0.3, and 0.5 g L^−1^. The range of tested concentrations was reduced due to the limited solubility of the ionic liquid in a 37.5 wt% sulfuric acid solution. The corrosion of the grid in the reference electrolyte, without the addition of ionic liquid, was also examined. The electrodes were polarized using the galvanostatic technique with a current density of 6 mA cm^−2^ for 10 days without a voltage limit. The acid temperature was maintained at 75 ± 2 °C. After exposure to the highly corrosive environment, the grids were etched with a solution of sodium hydroxide, hydrazine, and mannitol to remove the corrosion products layer. The weights of the grids were checked before and after the corrosion test.

### 2.3. Production of SLI type lead–acid batteries

All production stages were carried out in the Autopart company. Grids for positive and negative electrodes were obtained by the expanded metal method. For both electrodes, lead alloys identical to those from weight loss method studies, were used. The preparation of active masses, pasting of grids, plate curing, and assembly were carried out according to the standard procedures of the company. The battery cells were filled with a 33 wt% sulfuric acid solution with the ionic liquids additives at concentrations of 0.1, 0.3, and 0.5 g L^−1^. Reference cells (without ILs) were also filled with the same electrolyte. The resulting batteries were subjected to formation process using the Autopart company algorithm. This induce sulfuric acid concentration increase to 37.5 wt%. The battery specifications are shown in Table 1.

**Table 1:** Specification of the tested SLI lead–acid batteries

Parameter	Value
Voltage	12 V
Nominal capacity *C*_*n*_	55 A h
Electrolyte density	1.28 g cm^−3^

### 2.4. Battery tests

The battery tests were carried out in accordance with the European standards for starter batteries EN 50342:1 and EN 50342:6.^30,31^ The procedure sequences for each battery are presented in Table 2 and following subsections, while some of the detailed explanations are given in the ESI.† The tests were performed using battery testers DIGATRON UBT30-0/18-5M, DIGATRON UBT 10-18-64 HD, and DIGATRON HEW2000-12. Prior to each test, all batteries were charged in a water bath at a temperature 25 °C for 24 hours with a current 13.75 A, *i.e.* five times the nominal current *I*_*n*_ with a voltage limit 16.00 V. The use of nominal current values is also explained in the following subsections, ESI† and in Table 3, depicting required battery parameters.^30,31^

**Table 2:** Sequence of battery tests

Stage	Test	Battery no				
		1	2	3	4	5
1	Pre-testing charging	×	×	×	×	×
2	Capacity check	×	×	×	×	×
3	Cranking performance	×	×	×	×	×
4	Plate surface analysis	×				
5	Dynamic charge acceptance (DCA)		×			
6	Corrosion resistance			×		
7	Water consumption				×	
8	Cyclic durability at 50% depth of discharge (DoD)					×

**Table 3:** Set of required parameter values for SLI (55 A h) lead–acid batteries^30,31^

Parameter	Value
Effective capacity *C*_e_ (≥0.95*C*_*n*_), (20 hour capacity *C*_20_)	≥ 52.25 A h
Cranking current (CC)	550 A
Water loss	≤ 8 g A^−1^ h^−1^ (42 days)
Corrosion test	≥ 4 unit
Cycle life (50% DoD)	≥ 80 cycles
DCA	≥ 0.1 A A^−1^ h^−1^

#### 2.4.1. Battery capacity test^30^

The effective capacity of the battery (*C*_e_) is determined as the 20 hour capacity (C_20_) and is equal to the electrical charge that the battery can deliver at the discharge current (*I*_20_) to the final voltage *U*_f_ = 10.50 V. The nominal capacity (*C*_*n*_) of the tested batteries was 55 Ah. The *I*_20_ current, also referred to as *I*_*n*_ later on, was calculated using the formula:1
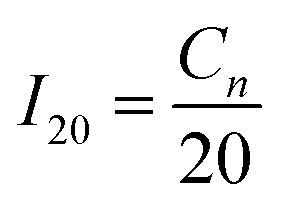


As a result, *I*_20_ = 2.75 A. The tests were conducted in a water bath at a temperature of 25 °C. The *C*_20_ capacity was calculated using the formula:

2 *C*_20_ = *t* × *I*_20_

where *t* [h] is the discharge time.

#### 2.4.2. Cranking performance test^30^

The test consists of two stages and involves determining the ability of the starting battery to deliver the nominal cranking current (*I*_cc_) for a period of 10 seconds at a temperature of −18 °C, followed by an additional 10 seconds of rest and then the ability to deliver a current of 0.6*I*_cc_ until a voltage of 6 V is reached. In the first stage, the required final voltage after 10 seconds (*U*_10s_) should not be less than 7.50 V. In the second stage, the time to discharge to 6 V (*t*_6v_) should be equal or higher than 90 s.

#### 2.4.3. Physicochemical and morphology analysis of lead–acid battery plates

The surface morphology and elemental composition of the active electrode masses and current collectors were estimated using a scanning electron microscope (SEM) Quanta 250 FEG with energy-dispersive X-ray spectroscopy (EDS) function.

#### 2.4.4. Dynamic charge acceptance (DCA) test^31,32^

The aim of the DCA test is to estimate the battery's ability to absorb peak current (*I*_DCA_) values at different levels of SOC (state of charge) after charging or discharging steps, as well as simulated “Stop–Start” (engine shutdown and restart) and regenerative braking operations. The whole sophisticated procedure is described in ESI.†

#### 2.4.5. Corrosion test^30^

The corrosion resistance test involves estimation of the durability of the battery under overcharging conditions at an elevated temperature. During one test cycle, batteries are placed in a water bath at a temperature of 60 °C and charged at a constant voltage equal to 14.00 V for 13 days. For the next 13 days, the battery is kept at the same temperature under open circuit conditions. After this time, bath is cooled to a temperature of 25 °C, while the battery is charged for 6 hours with a current equal to 13.75 A (5*I*_*n*_) and a voltage limit of 16.00 V. After 20 hours (open circuit conditions), batteries are discharged for 30 seconds with a current of 330 A (0.6*I*_cc_). Devices which, in the 30th second of the test, showed a voltage equal or higher than 7.2 V, reach the requirements for one cycle and proceed to the next one, carried out in the same way as described above. The requirement of the SLI battery standard is a minimum of 4 cycles performance.

#### 2.4.6. Water consumption test^30^

Water consumption test evaluates the performance of the battery under conditions of excessive heat and overcharging. Water consumption is determined as the loss of mass of a fully charged battery under overcharging conditions and is expressed in g A^−1^ h^−1^ for capacity *C*_e_. The test duration (42 days) corresponds to the requirements of the W3 and W4 standards, for which water consumption is less than 8 and 4 g A^−1^ h^−1^ for *C*_e_, respectively. Fully charged (according to the scheme in section 2.6) batteries were weighed and then placed in a water bath at 60 °C, and then charged at a constant voltage 14.40 V for 42 days. After this time, the battery was cooled to 25 °C, dried, and weighed again. The water consumption per ampere-hour of battery capacity was determined based on the weight difference:3
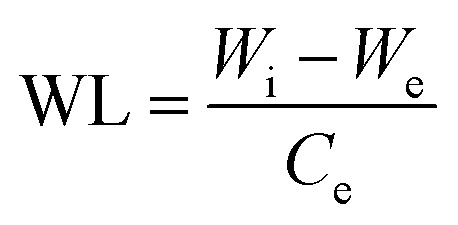
where WL represents the loss of battery mass, *W*_i_ and *W*_e_ depict the initial and final (at the end) battery weight, respectively.

#### 2.4.7. Endurance in cycle test with 50% depth of discharge (DoD)^31^

The batteries were placed in a water bath at 40 °C. The test cycle was consisted of discharging with a current *I*_dch_ equal to 13.75 A (5*I*_20_) for 2 hours, followed by charging to a value of 1.08 charging ratio (CR). The value of this ratio corresponds to the charging capacity *C*_rch_ calculated from the formula:4
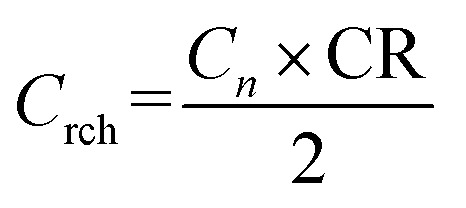


As a result, *C*_rch_ = 29.7 A h. Charging was carried out for a maximum 5 hours with a current equal to *I*_dch_ and a voltage limit 15.6 V. If CR was not reached, the batteries were charged additionally for a maximum 1 hour with a current *I*_20_ without a voltage limit until CR was reached. After the specified charging time or after delivering a charge of 29.7 A h, the next cycle started. If the voltage during discharge dropped below 10.0 V, the test was interrupted and the number of cycles was recorded.

### 2.5. Three-electrode electrochemical studies

Electrochemical studies in three-electrode cell systems were performed in order to understand the nature of the phenomena occurring in the lead–acid battery. Therefore, an alloy composed of lead, calcium, aluminum and tin was used as the working electrode. This is the material, which was utilized for the production of positive current collectors (grids) of a previously tested lead–acid batteries. The counter electrode was made of lead (99.9%), while the reference was a mercury/mercurous sulphate electrode (Hg|Hg_2_SO_4_ in 1 M H_2_SO_4_). The lead alloy and lead were produced by Autopart Company. The tested electrode was in the shape of a disk with a surface area of 4.7 cm^2^. The surface of the counter electrode was doubled to ensure efficient charge transfer. Electrochemical measurements were carried out in a solution of sulfuric acid (37.5 wt%) with the addition of ammonium ionic liquids at a concentration of 0.5 g L^−1^. In order to stabilize open circuit potential (ocp), the samples were initially kept in the electrolyte solution in an open circuit conditions for 4 h. Then, electrochemical impedance spectroscopy (EIS) measurements were performed in the frequency range from 10 mHz to 100 kHz (amplitude ±10 mV *vs.* ocp). After that, the potentiodynamic polarization (PP) tests were carried out in the potential range of −250 mV to +250 mV *vs.* ocp. Electrochemical experiments were carried out at ambient conditions using a VMP3 BioLogic® multichannel potentiostat/galvanostat with an impedance test module and EC-Lab® software.

## 3 Results and discussion

### 3.1. NMR, elemental and thermal analysis of ionic liquids

The results of NMR spectroscopy analysis for the investigated ionic liquids are presented below in the form of raw data, while graphs (Fig. S1–S12†) are provided in ESI.† For the compound di(hexadecyldimethylammonium) sulphate (marked as C16), peaks were recorded at the following concentration values (ppm): ^1^H NMR: 0.84–0.87 (t, 6H); 1.24–1.26 (m, 52H); 1.57–1.59 (m, 4H); 2.75 (s, 12H); 2.98–3.02 (m, 4H) and ^13^C NMR: 13.94; 22.11; 23.76; 25.90; 28.57; 28.74; 28.88; 29.00, 29.04; 29.09; 31.32; 42.28; 56.75. The results for di(octadecyldimethylammonium) sulphate (marked as C18) are presented as follows: ^1^H NMR: 0.84–0.87 (t, 6H); 1.19–1.24 (m, 60H); 1.54–1.58 (m, 4H); 2.70 (s, 12H); 2.92–2.96 (m, 4H) and ^13^C NMR (75 Hz, CDCl_3_): 14.00; 22.13; 23.99; 25.92; 28.56; 28.73; 28.85; 28.98, 29.07; 31.32; 42.28; 56.89.

As it can be seen, the structures of both compounds were confirmed. Protons from alkyl substituents were recorded as signals in the range of 0.84 to 0.87 ppm for methyl groups. Protons in methylene groups at the end of the alkyl chain were identified as peaks between 1.19 and 1.26 ppm. Methylene groups in the β position to the nitrogen atom appeared as signals from 1.54 to 1.59 ppm, while protons in the α position were noted in the range of 2.92 to 3.02 ppm. Methyl groups directly substituted to the nitrogen atom were identified as high peaks in the range of 2.70 to 2.71 ppm.

The results of the elemental analysis for both compounds are also evaluated: C_36_H_80_N_2_O_4_S (*M* = 637.10 g mol^−1^): calcd: C = 67.87; H = 12.66; *N* = 4.40; found: C = 68.11; H = 12.89; *N* = 4.16; C_40_H_88_N_2_O_4_S (*M* = 685.15 g mol^−1^): calcd: C = 70.12; H = 11.76; *N* = 4.09; found: C = 70.51; H = 12.08; *N* = 4.39.

**Fig. 1:**
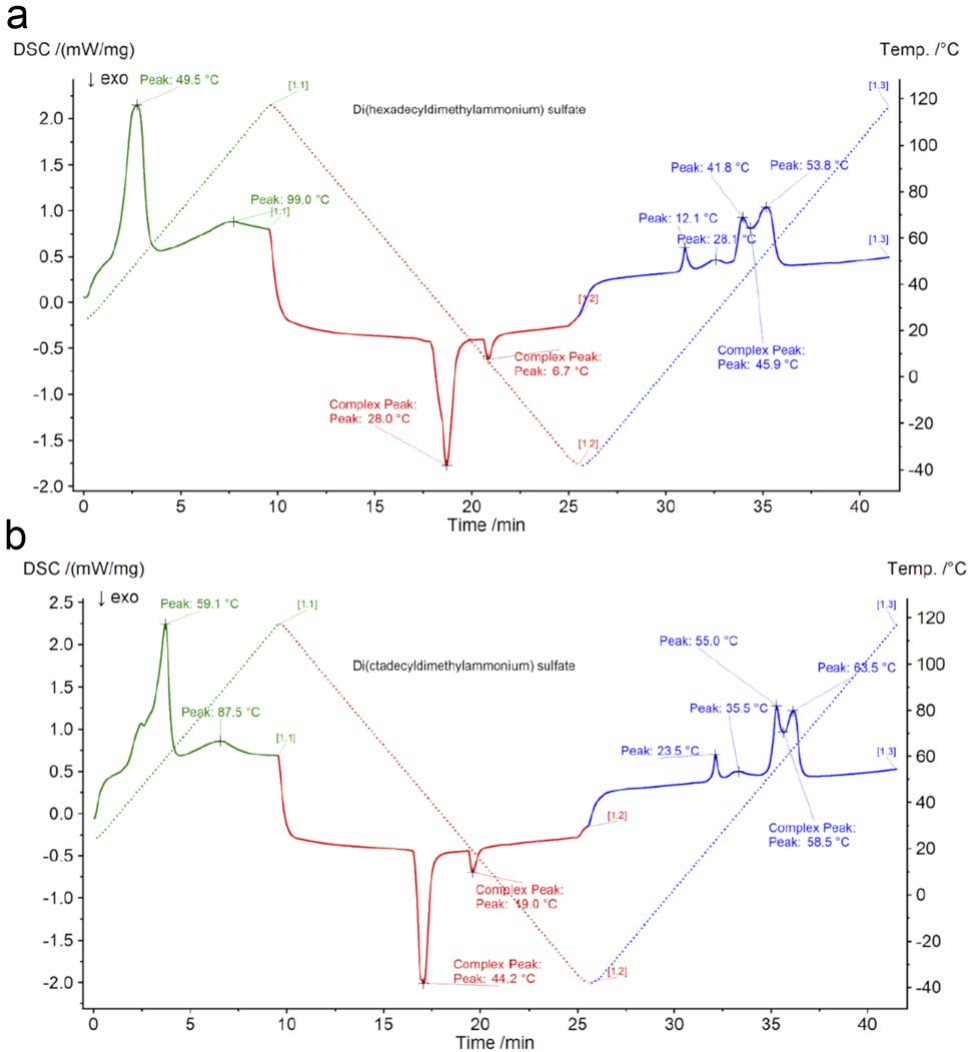
Thermal transition temperatures for (a) di(hexadecyldimethylammonium) sulphate and (b) di(octadecyldimethylammonium) sulphate.

Thermal analysis of di(hexadecyldimethylammonium) and di(octadecyldimethylammonium) sulphates (Fig. 1(a and b)) confirmed that the compounds can be referred to as ionic liquids, since their melting temperatures were respectively 53.8 °C and 63.5 °C, which is below 100 °C. The temperatures at which a 5% sample mass loss was observed were respectively 154 and 160 °C. This is a value significantly above the temperature range of lead–acid battery operation. Therefore, the investigated compounds are thermally stable enough considering their potential future applications.

### 3.2. Weight loss corrosion test of current collectors

**Fig. 2:**
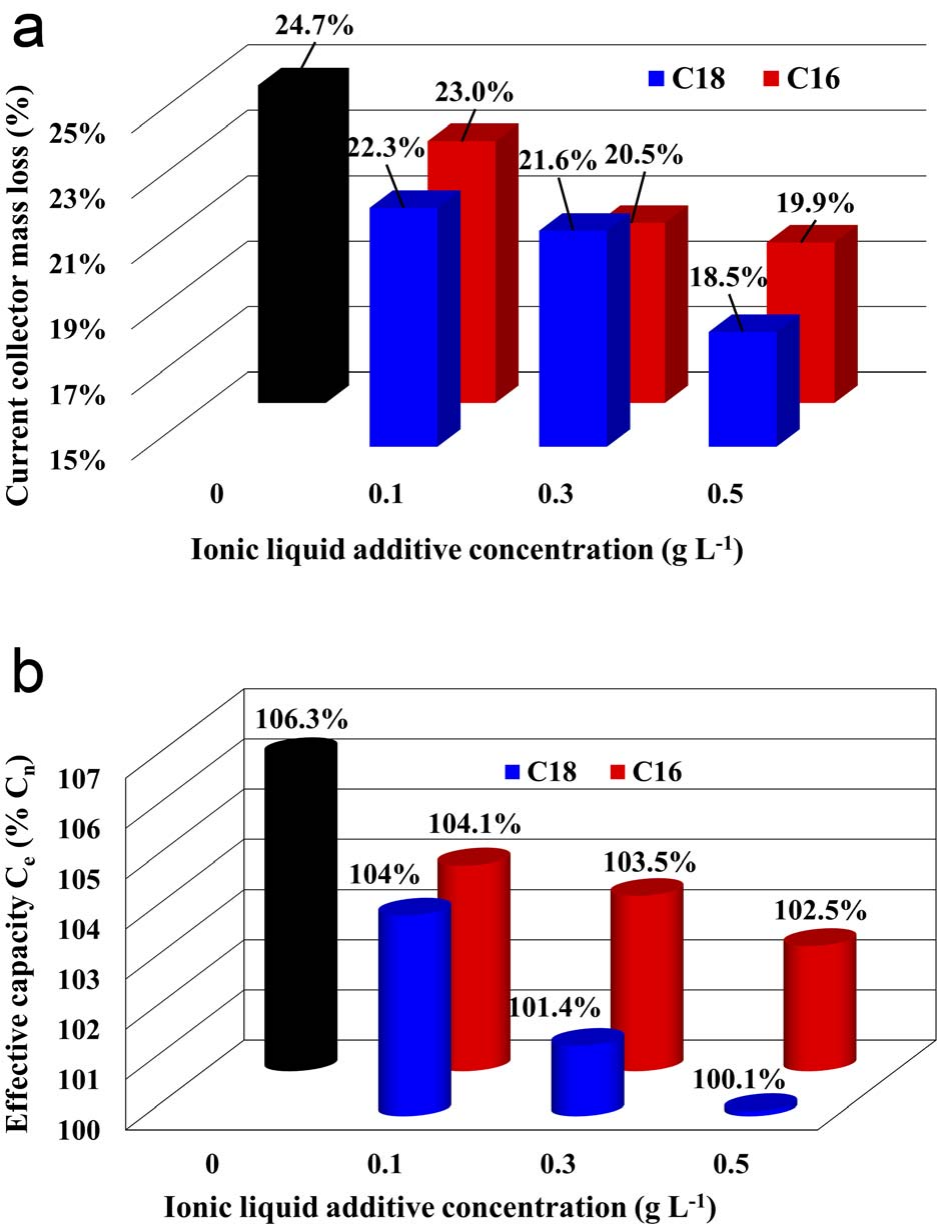
(a) Weight loss of current collectors after corrosion test in unmodified (black column) and ionic liquids modified (red and blue columns) sulfuric acid electrolytes. (b) Capacity check test results of batteries containing unmodified (black column) and ionic liquids modified (red and blue columns) sulfuric acid electrolytes.

The results of corrosion tests of grids (current collectors) of lead–acid batteries are presented in Fig. 2(a). The mass loss of the current collector in the electrolyte without the ionic liquid additive was 10.3 g, which corresponds to 24.7% of the initial value, *i.e.* mass before the test. All samples with the ionic liquid showed reduced mass loss, and an increase in the concentration of the additive caused an increase in this effect. In the case of di(octadecyldimethylammonium) sulphate compound, a greater positive impact was demonstrated in comparison to di(hexadecyldimethylammonium) sulphate. Therefore, the sample immersed in the electrolyte with the addition of 0.5 g L^−1^ of C18 compound achieved the most satisfying value, lower by the *c.a.* 6.2% than the current collector immersed in the unmodified electrolyte. The reason for the obtained results is explained in detail in subsections 3.3.3. and 3.4 while describing the results of physicochemical, morphological and three-electrode electrochemical tests.

### 3.3. Battery tests

Capacity check, cranking performance, DCA, corrosion resistance, water consumption, and cyclic durability tests were carried out in accordance with European standards.^30,31^ These standards are applicable to SLI batteries and their aim is to determine the requirements, essential functional characteristics, and appropriate test methods. Determining the operational parameters of the battery using standard tests is crucial in order to confirm the obtained effects.

#### 3.3.1. Battery capacity

The results of battery capacity tests are presented in Fig. 2(b). The capacity of the reference system (without IL) was 58.5 A h, which is 106.3% of the nominal capacity for *I*_20_ current. All batteries with the addition of an ionic liquid showed lower capacity compared to batteries without the addition. It was also observed that the increase in concentration causes greater loss of initial *C*_e_. For the lowest investigated concentration (0.1 g L^−1^), the influence of both tested ionic liquids was similar. At higher concentrations, a slightly more negative effect of di(octadecyldimethylammonium) sulphate was observed, reaching up to 5.8% loss for a concentration of 0.5 g L^−1^ in comparison to the reference system. Nevertheless, all batteries met the requirements of the standard regarding effective capacity (*C*_e_ > 0.95*C*_*n*_). During the discharge reaction of the battery, lead(ii) sulfate is formed on the negative electrodes. However, this compound is not formed on the lead surface directly after oxidation, but by diffusion of Pb^2+^ ions that migrate into the solution bulk. PbSO_4_ crystals precipitate on each other creating a porous structure. The incorporation of ionic liquid into this structure decrease the rate of the Pb^2+^ ions diffusion, which in turn cause a slight decrease in capacitance during the C20 test. Completely limiting diffusion would result in a drastic decrease in capacitance. The presence of ionic liquid molecules in the mass of lead(ii) sulphate is confirmed by the results of physicochemical tests, which are presented in subsection 3.3.3.

#### 3.3.2. Cranking performance test

The cranking performance test determines the ability of an SLI battery to start a combustion vehicle engine at low temperatures. The test results are presented in Table 4. The voltage of the battery without the addition of ionic liquid after 10 s of 550 A discharge was 7.85 V, and after 10 seconds of rest, the discharge to 6 V with 330 A current lasted 101 seconds. These results reach the requirements of the standard and confirm the correct manufacture of the batteries according to the Autopart company technology. However, batteries with ionic liquids showed lower values than those without the addition in both stages of the test, *i.e.* voltage and discharging time. It was also observed that with an increase in the concentration of ILs, the values of *U*_10s_ and *t*_6V_ decrease. The influence of both tested compounds was similar. All of the batteries met the standard requirements in both stages of the test (*U*_10s_ ≥ 7.50 V and *t*_6v_ ≥ 90 s).

**Table 4:** Cranking performance test results for batteries with and without the addition of ionic liquids in the sulfuric acid electrolyte

Ionic liquid	Concentration, g L^−1^	*U*_10s_, V	*t*_6v_, s
—	—	7.85	101
Di(hexadecyldimethylammonium) sulphate	0.1	7.83	99
	0.3	7.81	97
	0.5	7.79	96
Di(octadecyldimethylammonium) sulphate	0.1	7.80	98
	0.3	7.81	98
	0.5	7.77	97

#### 3.3.3. Physicochemical and morphology analysis of lead–acid battery plates

**Fig. 3:**
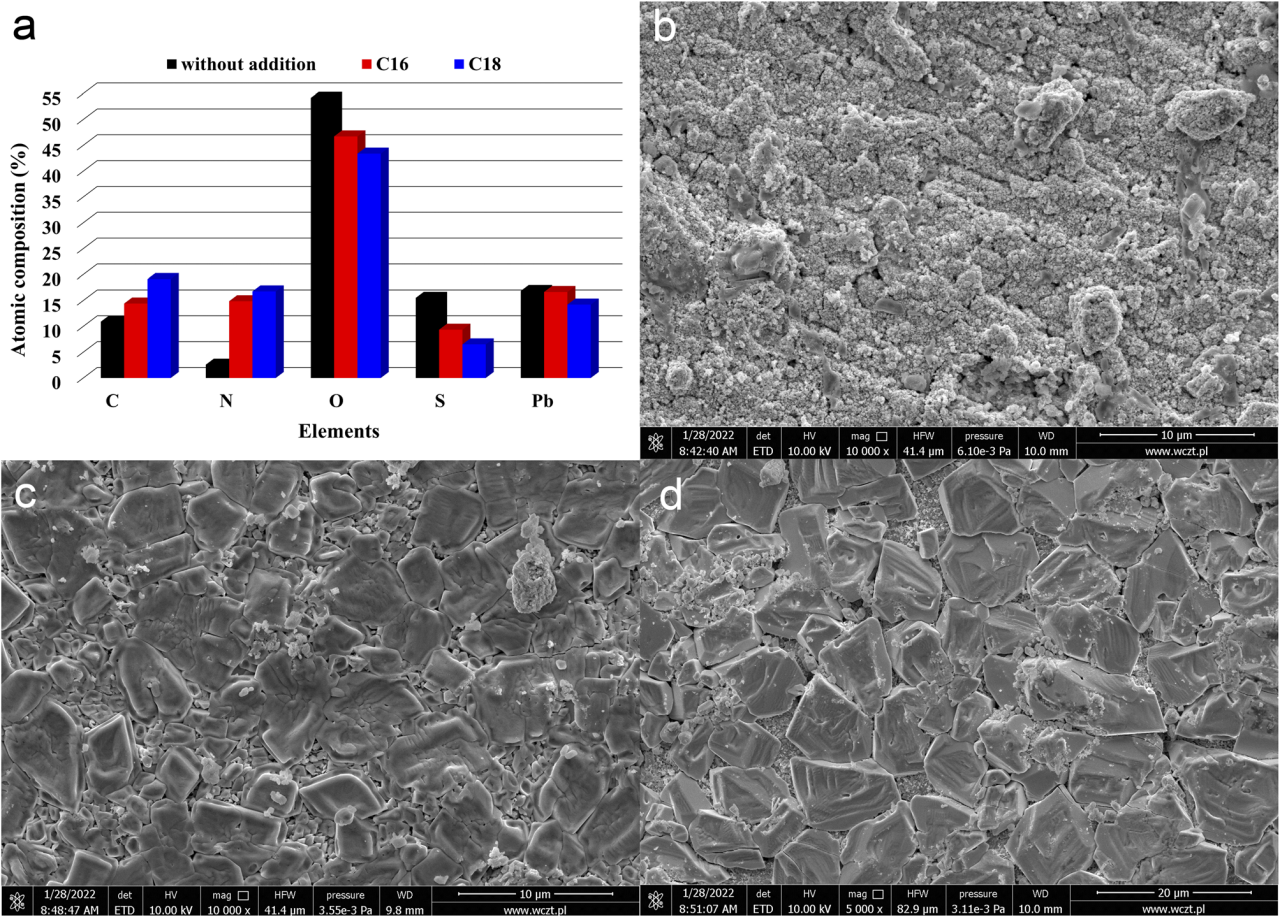
Chemical composition (a) and SEM images (b–d) of positive current collectors, derived from batteries containing unmodified (b) and ILs-modified ((c) C16, (d) C18) sulfuric acid electrolytes. Results represents the state after electrodes soaking, formation process and stages 1–3 of battery testing (Table 2).

The results of EDS analysis and SEM micrographs of the positive and negative current collectors surfaces are presented in Fig. 3(a–d). These results concern the components from reference and ILs-containing (0.5 g L^−1^) batteries. It should be noted that the above analysis refers to electrodes areas that had direct contact with the electrolyte solution, *i.e.*, they were not covered by the active mass. The samples were taken from cells manufactured according to Autopart company procedures and they were subjected to electrolyte soaking, formation process, C_20_ check test and cranking performance test. All this time, lead alloy continuously corrodes in an acidic environment, forming a layer of lead(ii) sulphate. A layer of lead(ii) oxide is also present, which forms even before placing the alloy in the electrolyte solution. Despite only two discharge and charge cycles associated with basic testing (Table 2, stages 1–3), corrosion products such as lead(ii) sulphate and lead oxide PbO_*n*_ (1 ≤ *n* ≤ 2) were visible on the current collector surface of the positive electrode in the battery without IL (Fig. 3(b)).^33–35^ The surface of the grid was degraded, with numerous cracks. Lead(ii) sulphate crystals were evenly distributed, and surface composition results confirmed mainly lead, sulfur, and oxygen content (Fig. 3(a)). It is obviously associated with exothermic soaking phenomena and strong, long-term polarization during formation and stages 1–3 of testing. However, on the current collectors from batteries with the addition of ionic liquid, there were no such tremendous signs of corrosion (Fig. 3(c and d)). In the case of modified electrolytes, molecules of ionic liquid containing long aliphatic chains are woven into the resulting sulphate and oxide layer, diminishing the rate of its creation. Moreover, the formation of a corrosion products, containing ILs compounds is confirmed by EDS analysis results (Fig. 3(a)). There was a significant decrease in oxygen and sulfur content in/on the current collector bulk/surface, while the presence of carbon and nitrogen increased, which unequivocally confirms the presence of an organic moieties. The effects caused by the tested compounds were similar, but di(octadecyldimethylammonium) sulphate slightly more effectively prevents the formation of lead(ii) sulphate and lead oxide particles. Moreover, as mentioned earlier, larger amounts of nitrogen and carbon were detected on the alloy surface (and subsurface). The formation of a protective layer on the positive electrode current collector surface explains the increase in its corrosion resistance and durability. The limitation in the formation of lead(ii) sulphate and lead oxide corrosion layers, which impede the transport of electric charges, explains the increase of dynamic charge acceptance capacity, especially in a partially discharged battery state, as indicated in the next subsection. Additionally, the obtained results of physicochemical tests are consistent with the results of weight loss corrosion method of current collectors, presented in subsection 3.2. The collectors were polarized positively (anodically), and therefore lead oxidation reactions to lead(ii) sulphate were forced on their surface. Collectors of positive electrodes from inside the battery were subjected to physicochemical tests, which does not change the fact that they were also polarized anodically. Therefore, the results of both studies are compatible. The presence of ionic liquids inhibits the formation of lead(ii) sulphate, which is a corrosion product of the current collector in the sulfuric acid environment. An in-depth explanation of the effect of the presence of ionic liquids and differences in their structure on the inhibition of corrosion of the current collector is explained in subsection 3.4, concerning three-electrode electrochemical studies.

**Fig. 4:**
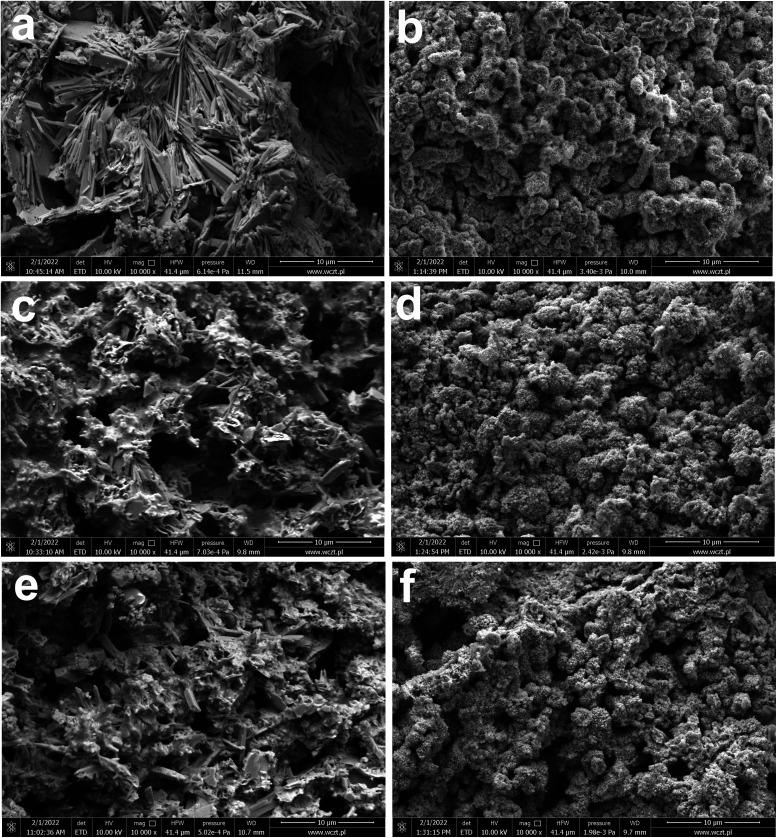
SEM images (a–f) of NAM (a, c and e) and PAM (b, d and f) from batteries containing unmodified (a and b) and ILs-modified ((c and d) C16, (e and f) C18) sulfuric acid electrolytes. Results represents the state after electrodes soaking, formation process and stages 1–3 of battery testing (Table 2).

In addition to current collectors, an analysis of the surface of active electrode materials, *i.e.* NAM (Negative Active Mass) and PAM (Positive Active Mass), was also performed. SEM images of samples from tested batteries are shown in Fig. 4(a–f). The samples for analysis were taken from the same batteries as above, *i.e.* previously subjected to soaking, formation and stages 1–3 of testing (Table 2). It has been shown that the addition of both tested ionic liquids affects the morphology of only NAM surface (Fig. 4(a, c and e)). Therefore, it seems that the applied ionic liquids affect the morphology and physicochemical properties of lead oxidation reaction products, *i.e.* lead(ii) sulphate. This is convergent with the above positive current collectors composition and morphology (Fig. 3(a–d)). Moreover, it was shown that the addition of both tested liquids does not cause any changes on the surface of PAM (Fig. 4(b, d and f)). Therefore, they do not influence reversible reaction of lead dioxide. Confirmation of the above conclusions and a broader analysis of the electrochemical phenomena were presented in the subsection describing the results of three-electrode electrochemical measurements.

#### 3.3.4. Dynamic charge acceptance test

**Fig. 5:**
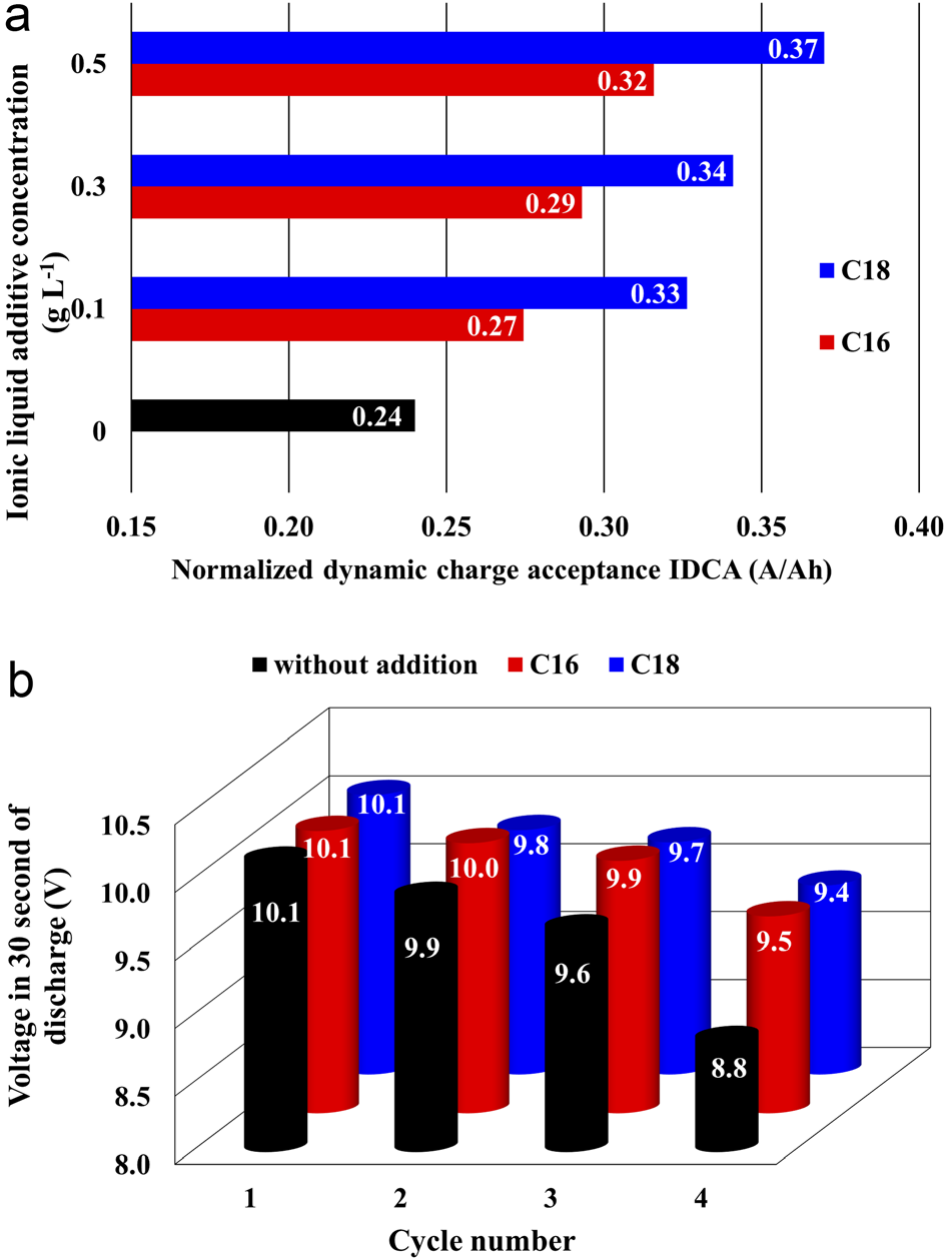
The normalized dynamic charge acceptance (DCA) capacity (a) and voltage value in 30 second of discharge during corrosion resistance test (b). The results concern lead–acid batteries containing unmodified (black) and ILs-modified (red and blue) sulfuric acid electrolytes.

The results of DCA tests are presented in Fig. 5(a). The *I*_DCA_ value for unmodified battery (without IL) was equal to 0.23 A A^−1^ h^−1^. All of the modified battery systems, containing C16 and C18 compounds are characterized by higher *I*_DCA_ parameter than those without the additive. It was also observed that with increasing IL concentration, there is an increase of *I*_DCA_ value. A more positive effect of di(octadecyldimethylammonium) sulphate (0.34 A A^−1^ h^−1^) was demonstrated at the applied concentration of 0.5 g L^−1^, which represents over 50% increase in DCA in comparison to the reference battery. Significant differences were also observed for both tested compounds in the *I*_d_ and *I*_r_ components corresponding respectively to the average charging current in a partially discharged state and average regenerative charging current, which also applies to the battery in a discharged state in cycles simulating standard battery use. The results obtained are fully consistent with the SEM and EDS analysis. Absorption of ionic liquids in the structure of the formed lead(ii) sulphate reduces the sulphation of the NAM and corrosion of the current collectors of the positive electrodes. This in turn reduces the resistance of the battery in the state of partial discharge, thus increasing the DCA.

#### 3.3.5. Corrosion resistance

The results of battery corrosion tests are presented in Fig. 5(b). All of the tested devices achieved the standard requirement and completed the full four test cycles. All batteries showed similar results after the first three cycles. After fourth one, the voltage values after 30 seconds of discharge of both batteries with added ionic liquids were higher by over 0.50 V in comparison to reference battery. The influence of both tested ionic liquids was similar and there was no significant effect of the additive concentration in the electrolyte solution. Battery without the IL additive failed during the charging stage after completing the fourth test cycle. The remaining systems failed during the overcharging stage at elevated temperature during fifth cycle. Therefore, it is concluded that the improvement in corrosion resistance test results is associated with the presence of ionic liquids, but the effect is not strong enough for the batteries to perform an additional cycle in the above test.

#### 3.3.6. Water consumption test

**Fig. 6:**
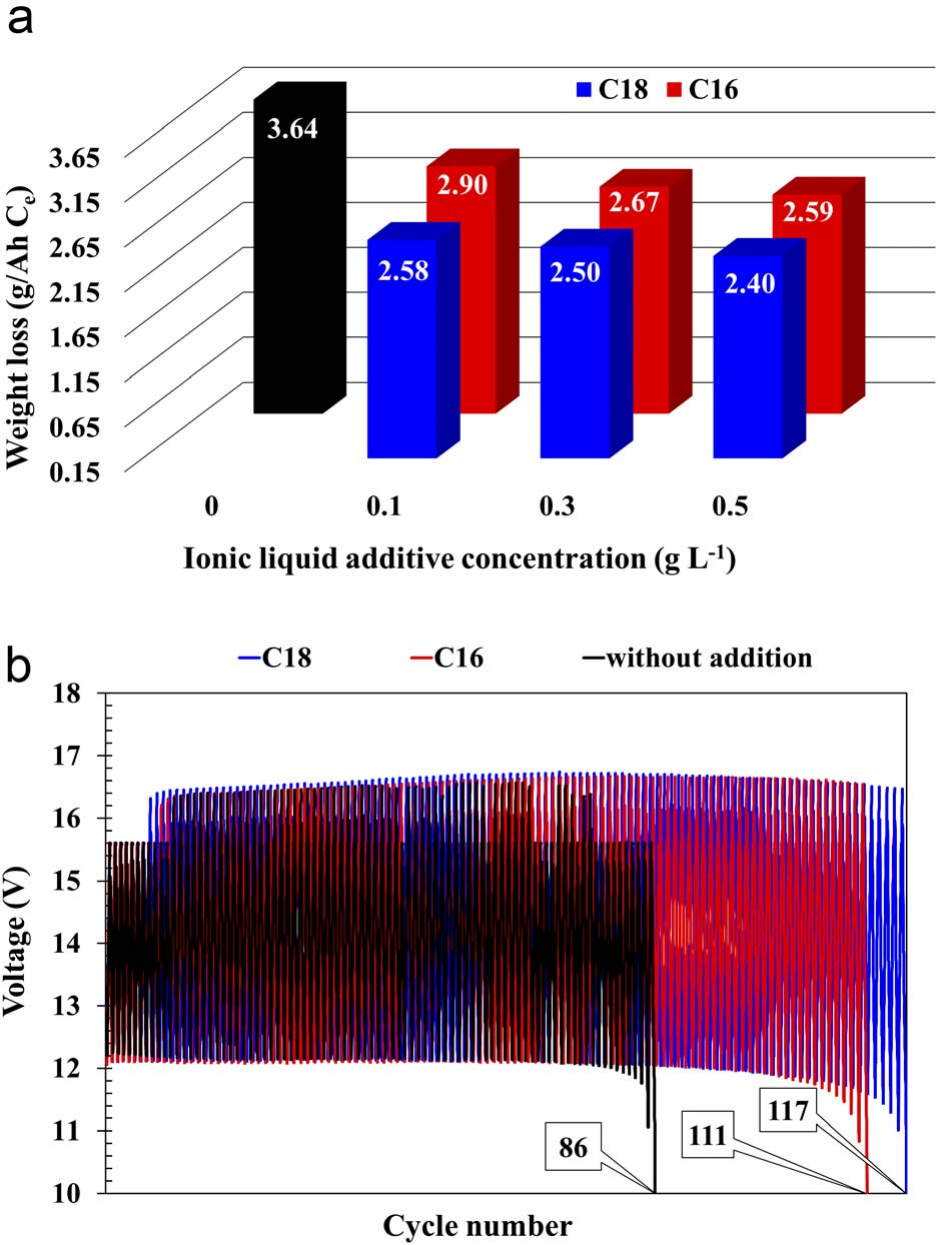
Water consumption, *i.e.*, battery weight loss (a) and cycling durability (b) tests results of batteries containing unmodified (black) and ILs-modified (blue and red) sulfuric acid electrolytes.

The water consumption of the battery without the addition of ionic liquid (Fig. 6(a)) was equal to 3.64 g A^−1^ h^−1^ for the *C*_e_ capacity. Therefore battery meets the requirements of the standard at W4 level. All batteries with the ILs additives, despite slightly lower *C*_e_ values, showed reduced water consumption. Moreover, higher concentration of ionic liquids increases this effect. The lowest water consumption was achieved by the battery with the addition of 0.5 g L^−1^ di(octadecyldimethylammonium) sulphate, reaching 2.40 g A^−1^ h^−1^, which represents a 34% decrease in comparison to the battery with unmodified electrolyte solution.

#### 3.3.7. Endurance in cycle test with 50% depth of discharge

The cyclic endurance test involves estimation of the battery's ability to perform repeated charge/discharge cycles to only 50% of its nominal capacity. During cycling, the reference cell voltage decreased to 10.0 V after 85 cycles (Fig. 6(b) and Table 5).

**Table 5:** Endurance in cycle test with 50% depth of discharge for batteries containing unmodified and ILs-modified sulfuric acid electrolytes

Ionic liquid	Concentration g L^−1^	Cycles	
		No.	%_Ref_
—	—	85 (_Ref_)	100%
C16	0.1	99	116%
	0.3	102	120%
	0.5	109	128%
C18	0.1	110	129%
	0.3	113	133%
	0.5	116	136%

All batteries with the modified electrolytes indicated increased cyclic durability, and an increase in the concentration of the IL additive extends their lifetime (Table 5). Moreover, the battery with the highest C18 concentration reached 116 cycles, which is a 36% increase when compared to the reference system. Higher cyclic endurance of the electrolyte-modified devices is the result of the findings of all previous studies, *i.e.*, increased corrosion resistance of the current collectors (3.2), increased DCA (3.3.4), and reduced water consumption (3.3.6).

### 3.4. Three-electrode electrochemical studies

The three-electrode electrochemical analysis was carried out for three electrolyte solutions, *i.e.* sulfuric acid and sulfuric acid with the addition (0.5 g L^−1^) of the two above tested ionic liquids. This specific concentration had the most significant impact on improving the operating parameters of the batteries, therefore it was also chosen for three-electrode analysis. Based on the research on lead–acid batteries presented in the previous part of the manuscript, it should be mentioned that the increase in concentration causes a decrease in the solubility of the ionic liquid and formation of foam since these compounds act as a surfactants. Therefore, 0.5 g L^−1^ was the highest possible applied concentration.

**Fig. 7:**
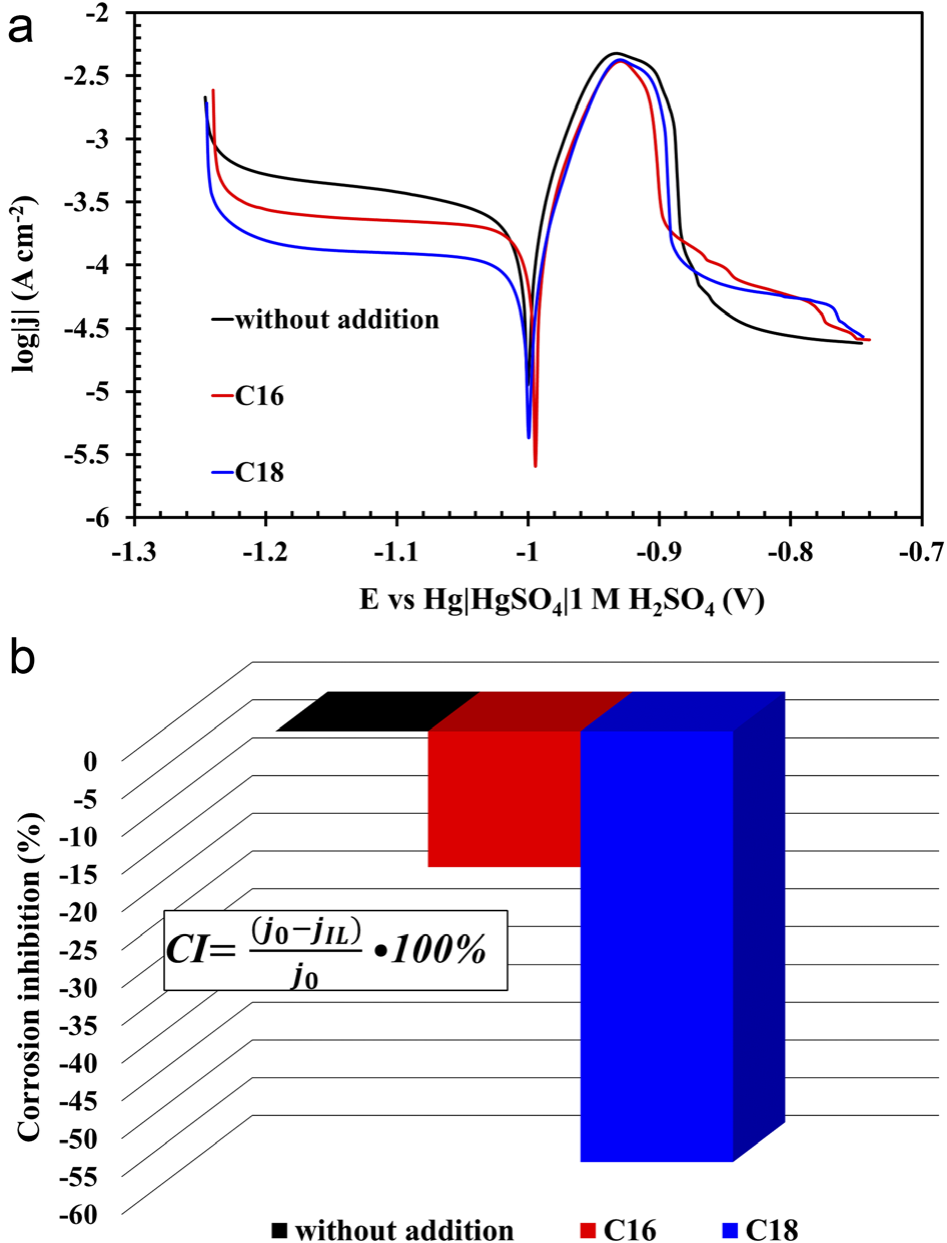
Potentiodynamic polarization curves (a) and corrosion inhibition (CI) values (b) of Pb–Ca–Sn–Al alloy in unmodified (without ILs addition) and ILs-modified 37 wt% sulfuric acid electrolyte. Corrosion inhibition (CI) values were calculated from corrosion current densities (*j*_0,IL_) derived from the curves (*j*_0,IL_ stands for unmodified and ILs-modified electrolyte, respectively).

According to the PP curves (Fig. 7(a)), there are no significant differences between corrosion potentials (*E*_corr_), however, the corrosion current density values (*j*_corr_) of the lead alloy samples in modified electrolytes significantly decreased (Fig. 7(a)). Therefore, it can be concluded that the presence of both tested compounds in the solution of sulfuric acid inhibits the rate of lead corrosion (CI – corrosion inhibition, Fig. 7(b)). The improvement of the corrosion resistance of the Pb–Ca–Sn–Al alloy is related to the absorption of ionic liquid molecules into the lead(ii) sulphate layer that is formed in sulfuric acid. The presence of this layer was also demonstrated in physicochemical tests (SEM and EDS) (3.3.3) of the grids and NAM samples. It is worth noting that near the corrosion potential, oxidation and reduction reactions on the surface of the tested electrode proceed at the same rate. This is the rate determined by the slower process. In this case, it is a reduction, as indicated by the cathodic polarization curves. Their slopes are higher in relation to the ordinate axis than that of the anodic. In addition, the presence of ILs in the electrolyte solution inhibits the reduction reaction to a much greater extent than the oxidation one. The anodic current density values are slightly higher during lead polarization in the unmodified electrolyte than the values in the case of ILs-modified electrolytes. However, these differences are much more pronounced in the case of cathodic polarization. At this point, it is worth recalling once again that the polarization test was carried out after several hours of lead presence in a 37 wt% solution of sulfuric acid. The lead working electrode undergoes oxidation reactions while striving for the equilibrium state. Lead(ii) sulfate is then formed. Considering the fact that the solution is concentrated, hydrogen ions are reduced, of course on the surface of the lead working electrode. The presence of ILs in the electrolyte means that during the anodic polarization of the electrode, the current density values in the potential range from −1 V to approx. −0.88 V are lower compared to the electrode in the unmodified electrolyte. As a result, less amount of the lead(ii) sulfate is formed. Taking into account low conductivity values of PbSO_4_, the values concerning unmodified electrolyte rapidly decrease above the potential of *ca.* −0.88 V. The same applies to the phenomenon of cathodic polarization, and in this case lead(ii) sulfate, which is already produced at the open circuit conditions plays a significant role. Certainly, it covers to a lesser extent the electrodes in solution with ILs. During the cathodic polarization, the rate of hydrogen evolution increases and the reduction of lead(ii) sulfate to metallic lead begins. A lower amount of lead(ii) sulfate on lead electrodes causes the inhibition of reduction reaction. Hence, the lower current density values are recorded during the cathodic polarization. Comparing the two ILs used, the compound containing 18 carbon atoms in the aliphatic chain is more effective at inhibiting lead corrosion than its lighter counterpart. The use of di(hexadecyldimethylammonium) sulphate resulted in a decrease in the corrosion current density by 18%, and for di(octadecyldimethylammonium) sulphate by 57%. The difference seems to be extremely significant. The above observations are the result of the increase in the length of the aliphatic chain, which may explain the slightly worse anti-corrosive properties of di(hexadecyldimethylammonium) sulphate, which is also evidenced by weight loss corrosion test of current collectors.

**Fig. 8:**
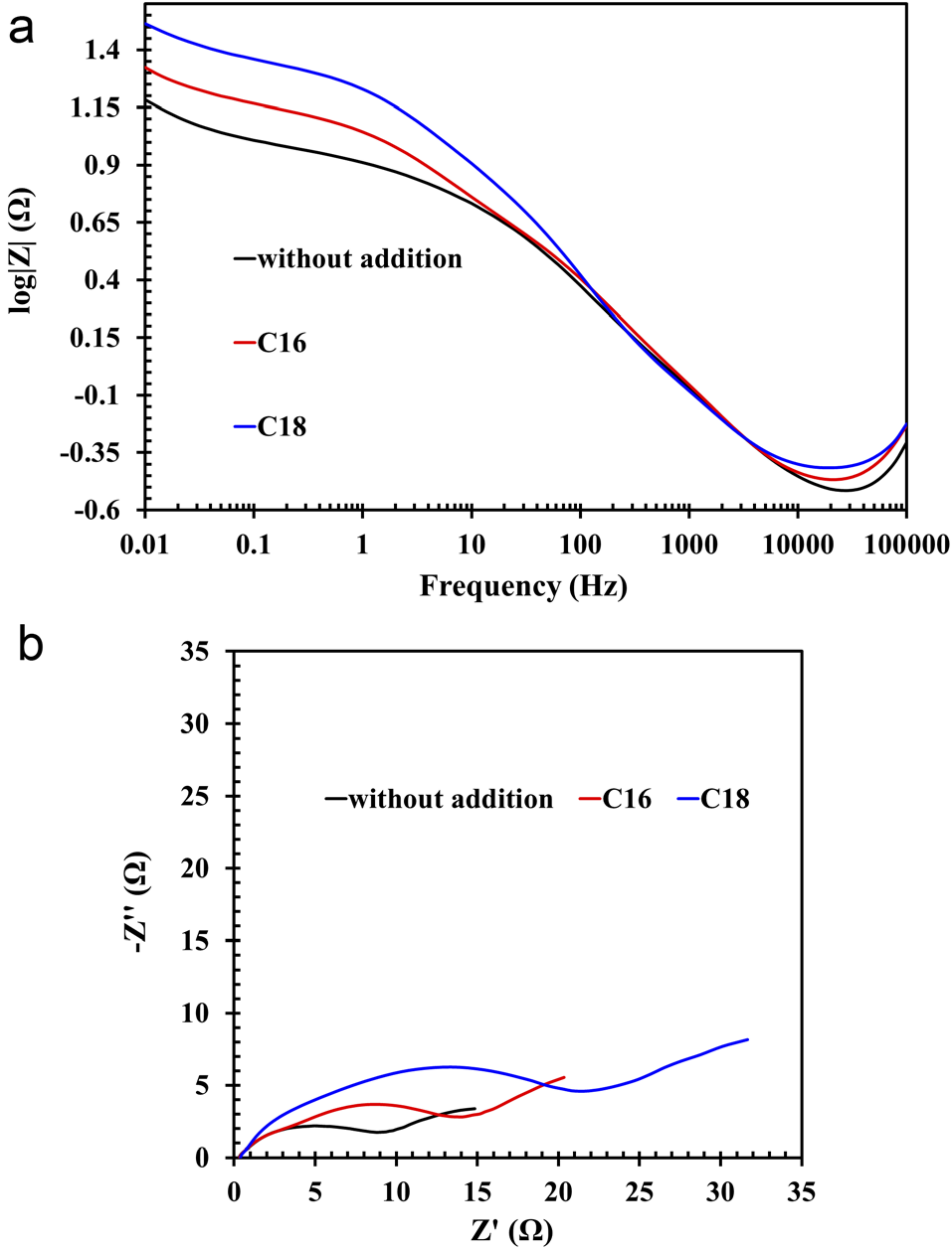
(a) Bode and (b) Nyquist plots of Pb–Ca–Sn–Al alloy in unmodified (without ILs addition) and ILs-modified 37 wt% sulfuric acid electrolyte.

All of the above theses are confirmed by the EIS measurements, depicted in the form of Nyquist and Bode curves (Fig. 8(a and b)). The nature of the former indicates the presence of three time constants (semi-circles) appearing sequentially in the range from the highest to the lowest frequency values.^36,37^ The first semi-circle is by far the smallest for the lead sample in unmodified solution. The impedance test was performed 4 hours after immersion of the lead in the electrolyte solution at open circuit conditions. All this time, the alloy strongly corrodes in an acid environment, thus a mentioned layer of lead(ii) sulphate is formed. There is also a layer of lead(ii) oxide that forms before the alloy is placed in the electrolyte solution. In the case of modified electrolytes, molecules of ionic liquid containing long aliphatic chains are woven into the resulting sulphate and oxide layers. Therefore the resulting film has better anti-corrosive properties. The presence of ILs compounds on the surface of lead alloy is also confirmed by the previously presented results of physicochemical analysis. The first time constant in the Nyquist curves, which is almost imperceptible for the unmodified sample, concerns the lead(ii) sulphate layer, which is in direct contact with the electrolyte solution. The next time constants are the lead(ii) oxide and the surface of the lead alloy. It was noticed that in ILs containing electrolyte solution, there was a rapid increase in the diameter of the first time constant, which confirms the absorption of the ionic liquid molecules by the emerging lead(ii) sulphate layer. The decrease of the corrosion phenomena rate in modified electrolyte is also indicated by the Bode curves, and more specifically the curves depicting dependence between impedance modulus and the frequency. In the range of the lowest frequency values, the highest impedance modulus values are characteristic for lead samples in solutions with the addition of ILs.

The absorption of ionic liquids causes an increase in the resistance of the entire system, which explains the lower values of capacity (Fig. 2(b)), voltage (Table 4) and discharged time (Table 4) at the beginning of the battery life in comparison to its counterpart without this additives. This does not change the fact that, in the long term, the presence of ionic liquids in the lead(ii) sulphate layer inhibits the growth of this layer on the surface of the current collector of the positive electrode and the growth of the active mass on the negative electrode. This is an effect of anti-corrosive properties of the created layer, which is enriched with long-chain ionic liquid molecules. This is evidenced by the results of weight loss corrosion test, lead grid three-electrode corrosion measurements and battery tests, including DCA, cyclic durability, corrosion resistance and water consumption.

## 4 Conclusions

Electrochemical studies have confirmed the effectiveness of inhibiting the corrosion of Pb–Ca–Sn–Al alloy through modification of electrolyte composition with ionic liquids. Furthermore, the conducted research has also confirmed that the addition of these compounds to the electrolyte significantly affects the performance parameters of SLI batteries. The investigated ionic liquids are absorbed in the layer of lead(ii) sulphate, formed on the surface of current collectors and negative electrode active masses. Therefore, electrochemical corrosion of current collectors and sulfation phenomena of negative electrodes are inhibited. Electrical tests of the batteries have shown that the addition of these compounds improves the dynamic charge acceptance capability, reduces water consumption, and inhibits the corrosion of current collectors. All of the above leads to an increase in their service life. The obtained results confirm that the addition of ammonium ionic liquids to the electrolyte in lead–acid battery is a promising direction for improving the durability of these devices.

## Author contributions

Paweł Kędzior: investigation, methodology, formal analysis, validation, writing – original draft. Waldemar Rzeszutek: writing – review & editing. Jarosław Wojciechowski: conceptualization, methodology, validation, writing – original draft, visualization. Andrzej Skrzypczak: investigation, resources. Grzegorz Lota: supervision, review & editing, project administration, funding acquisition.

## Conflicts of interest

There are no conflicts to declare.

## Supplementary Material

RA-013-D3RA04386J-s001
