# Supplementary material for: Enhanced cycle life of starter lighting ignition (SLI) type lead–acid batteries with electrolyte modified by ionic liquid
Source: RSC Adv. 2023 Aug 7;13(34):23626–37. doi: 10.1039/d3ra04386j (PMC10405049; doi:10.1039/d3ra04386j)
Supplement: RA-013-D3RA04386J-s001 [file RA-013-D3RA04386J-s001.pdf]

Electronic Supplementary Material (ESI) for RSC Advances.
This journal is © The Royal Society of Chemistry 2023

SUPPLEMENTARY INFORMATION

Enhanced cycle life of starter lighting ignition (SLI) type lead-acid batteries with electrolyte modified by ionic liquid

Paweł Kędzior,[a] Waldemar Rzeszutek,[a] Jarosław Wojciechowski,*[b] Andrzej Skrzypczak[b] and Grzegorz Lota*[b, c]

[a.] *PPUH Autopart Jacek Bąk sp. z o.o., Kwiatkowskiego 2A, Mielec 39-300, Poland.*

[b.] *Institute of Chemistry and Technical Electrochemistry, Poznan University of Technology, Berdychowo 4, Poznań 60-965, Poland. E-mail addresses: grzegorz.lota@put.poznan.pl (G. Lota), jaroslaw.g.wojciechowski@put.poznan.pl (J. Wojciechowski).*

[c.] *Łukasiewicz Research Network – Institute of Non-Ferrous Metals Division in Poznan, Central Laboratory of Batteries and Cells, Forteczna 12, 61-362 Poznan, Poland.*

2. Experimental

2.1. Ionic liquids selection, synthesis and physicochemical analysis

The choice of di(hexadecyldimethylammonium) sulphate and di(octadecyldimethylammonium) sulphate was made taking into account several criteria. Firstly, research on the addition of di(octadecyldimethylammonium) sulphate has not appeared in the available literature so far, while di(hexadecyldimethylammonium) sulphate has been used as an additive to the active mass of the positive electrode.[26] However, the modification of the active material of the positive electrode with an ionic liquid results in the need for continuous and tedious control of the technological process of production of lead-acid batteries, while the modification of the electrolyte is a trivial technological task. In addition, the presented studies have shown that the elongation of the carbon chain of the cation also has a positive effect on the parameters of the battery. Based on the analysis of the literature, sulphate alkylammonium ionic liquids were selected as the research object. The basic assumptions for the structure of the compound were a cation containing a linear alkyl substituent consisting of a minimum of sixteen carbon atoms and a sulphate anion being an ion common to the electrolyte. In addition, it has been shown that the expansion of the cation structure has a positive effect on the corrosion parameters of the lead-acid battery system.[19] The increase in the length of the carbon chain of the cation also slows down the rate of corrosion.[23,24] The above-mentioned criteria are met, among others, by three groups of compounds: di(alkylammonium) sulphates, di(alkyldimethylammonium) sulphates and di(alkyltrimethylammonium) sulphates. Due to the planned research not only on a laboratory, but also on technological scale, the key criterion for the selection of these liquids was their solubility and surface effects in the form of foam formation on the surface of the electrolyte. Two groups of compounds were excluded from the study at an early stage. Di(hexadecyl ammonium) sulphate is sparingly soluble and forms a suspension in 37.5% sulfuric acid electrolyte. The introduction of ionic liquid in the form of a suspension into the battery cell makes it impossible to obtain homogeneity of this compound in the entire cell volume. Di(hexadecyltrimethylammonium) sulphate shows good solubility in 37.5% sulfuric acid electrolyte, but it causes strong surface effects even at minimal concentrations, causing electrolyte losses during the charging process due to intensive evolution of hydrogen and oxygen. Di(hexadecyldimethylammonium) sulphate showed good solubility and caused small surface effects and was selected for testing. By extending the main chain of this compound, di(octadecyldimethylammonium) sulphate was obtained, which did not show surface effects, but was less soluble. By appropriate selection of concentrations, it was also used for research. Further elongation of the carbon chain of a compound from this group would cause even more difficult solubility and probably the inability to use it in research, but it was not possible to verify these predictions due to the lack of availability of reagents for the synthesis of such a structure.

Reagents for the synthesis of organic ionic liquids (ILs), i.e. N-alkyl-N,N-dimethylamines (≥95%) with the general formula $(CH_3)_2HNR$, sulfuric acid (VI) (98%), and an organic solvent (ethyl acetate), were purchased from Merck (Merck Life Science) and used without further purification. A 0.02 mole portion of the amine was dissolved in 50 cm^3 of the solvent. The solution was vigorously stirred, and 0.02 moles of concentrated (96 wt.%) sulfuric acid were slowly added. The product precipitated from the solution as a white solid. After 15 minutes, the solvent and the water formed as a byproduct were evaporated and product was dried under vacuum at 60 °C. Using the above method, two compounds with the formulas $2[(CH_3)_2HNC_{16}H_{33}]^+ SO_4^{2-}$

(di(hexadecyldimethylammonium) sulphate) and $2[(CH_3)_2HNC_{18}H_{37}]^+ SO_4^{2-}$ (di(octadecyldimethylammonium) sulphate) were obtained.

^{1}H NMR spectra were recorded using a Varian Mercury 300 spectrometer operating at a frequency of 300 MHz, with tetramethylsilane (TMS) as the internal standard. ^{13}C NMR spectra were obtained using the same instrument at a frequency of 75 MHz.

The thermal transition temperatures of the ionic liquids were determined using Differential Scanning Calorimetry (DSC) with a Netzsch DSC 214 Polyma instrument operating under a nitrogen atmosphere. A sample of the ionic liquid (5-15 mg) was heated in the temperature range of 25 to 120 °C at a rate of 10 °C min^{-1}, then the sample was cooled to -100 °C at a rate of 10 °C min^{-1} and heated again to 120 °C. Thermogravimetric analysis was performed using a Mettler Toledo TGA/DSC1 instrument. The ionic liquids (2-10 mg) were placed in aluminum pan and heated from 30 to 450°C at a rate of 10°C min^{-1} under a nitrogen atmosphere.

2.2. Battery tests

2.2.1. Cranking performance test[30]

The batteries were placed in a cooling chamber for 24 hours until the internal temperature of the cells reached -18 °C. The test consists of two stages and involves determining the ability of the starting battery to deliver the nominal cranking current (I_{cc}) for a period of 10 seconds at a temperature of -18 °C, followed by an additional 10 seconds of rest and then the ability to deliver a current of 0.6 I_{cc} until a voltage of 6 V is reached. In the first stage, the required final voltage after 10 seconds (U_{10s}) should not be less than 7.50 V. In the second stage, the time to discharge to 6 V (t_{6V}) should be equal or higher than 90 s. The nominal cranking current of the tested batteries was I_{cc} = 550 A and such a current was used to discharged the batteries in the first stage of the test, while in the second stage it was 0.6 I_{cc} = 330.

2.2.2. Dynamic charge acceptance test[31,32]

The batteries were placed in a water bath at a temperature of 25°C. The DCA test consists of three consecutive parts. In the first part, the batteries underwent initial cyclic operation consisting of twice and then once discharging with currents respectively of 25 A and 2.75 A (I_{20}), for a period of 3 h or until the voltage of 10.50 V was reached (discharge 25 A) and for a period of 26 h or until reaching a voltage value of 10.50 V (discharge 2.75 A). After each discharge, the batteries were charged with a current of 13.75 A (5•I_{20}) and with a voltage limit of 15.60 V for 24 h. The last (third) charging stage lasted until the capacity value was reached:

$$C = C_{20} - 0.2 \bullet C_n \quad \text{(S1)}$$

Each subsequent discharge occurred 1 hour after the end of charging. In the second part of the study, which takes place 20 hours after the end of the first part, the batteries were subjected to two pulse tests. One test consisted of 20 following pulses: charging with a current of 91.57 A (33.3•I_n) for 10 seconds with a voltage limit of 14.80 V, a 30-second pause, discharging with a current of 55 A (20•I_n) for a time allowing to obtain the charge delivered during charging and next pause. After the 20th pulse, the batteries were charged for 12 hours with a current of 13.75 A (5•I_n) and voltage limit to 16.00 V and additionally for 4 hours with a current of 1.37 A (0.5•I_n) and voltage lower than 18.00 V. An hour after the end of charging, the batteries were discharged for 2 hours with a current 2.75 A (I_n), and after another 20 hours of pause, 20 charge/discharge pulses were performed again, as described above. After the pulse test, the batteries were discharged for 2 hours with a current 2.75 A. The start of the third part of the study occurs 12 hours after the end of the second part. In this case DCA is measured during simulated real vehicle operation with stop/start function. Before starting this stage, two resistors (681.8 Ω each) were connected in parallel to the battery terminals. The resistor simulates the vehicle's idle power consumption. Each of the 5 cycles of the study consists of several stages: (I) 12-hour pause, (II) engine start (turning the key in the ignition), which discharges the battery with a current of 2.75 A (I_n) for 30 seconds, (III) engine start (turning on the engine) (discharging with a current of 100 A for 3 s), (IV) battery charging (58 s, 91.57 A (33.3•I_n), 14.40 V), (V) the first internal cycle in order: 30 s discharging with a current of 3.44 A (1.25•I_n), 30 s charging with a current of 91.57 A with a voltage limit of 14.40 V, 30 s pause, (VI) regenerative braking, i.e., charging with a current of 91.57 A and voltage limit to 15.00 V for 5 s, (VI) activating the start/stop system, stop function: discharging with a current of 27.5 A (10•I_n) for 10 s, (VII) activating the start/stop system, start function: discharging with a current of 100 A for 1 s, (VIII) the second internal cycle in order: 20 s discharging with a current of 3.44 A (1.25•I_n), 20 s charging with a current of 91.57 A and voltage limit of 14.40 V, 20 s pause, (IX) look at the stage VI, (X) the third internal cycle in order: 20 s discharging with a current of 13.75 A (5•I_n), 20 s charging with a

current of 91.57 A and voltage lower than 14.40 V, 20 s pause. In only one cycle, stages (V) to (X) should be repeated 19 times in a cyclic manner. The course of each microcycle depends on the state of charge (SOC) of the battery. The decision algorithm during the loop of these stages prioritizes charging the battery and does not allow for further discharges. Each internal cycle simulates one day of driving. The stages that follow are: (XI) discharge of 5.5 A (2•I_n) for 30 s, (XII) discharge with 2.89 A (1.05•I_n) for 120 s, (XIII) discharge with 1.15 A (0.4182•I_n) for 330 s, (XIV) 3.33 h break. In one cycle, stages from (II) to (XIV) are repeated three times in a cyclic manner during the one complete cycle. Five cycles are performed, consisting of all the above stages.

The normalized dynamic charge acceptance capacity (I_{DCA}) is expressed in A/Ah and is calculated using the formula:

$$I_{DCA} = 0.512 \bullet \frac{I_c}{C_n} + 0.223 \bullet \frac{I_d}{C_n} + 0.218 \bullet \frac{I_r}{C_n} - 0.181 \quad (S2)$$

where: I_c is the average charging current for 20 pulses after the previous charging stage (part two of the study); I_d is the average charging current during 20 pulses after the previous discharge stage (part two of the study); I_r is the average regenerative charging current (part three of the study). I_c and I_d were calculated from the integrated amount of charge of all pulses, divided by the total charging time in the second part of the study. I_r was calculated by integrating the ratio of the charge delivered in all charging stages of the third part of the study to the total charging time. All calculations defined above were automatically performed during the test using the Digatron © Battery Manager Workstation software interface version 4.25.2.253 on the test bench according to the standard recommendations.

3. Results and discussion

3.1. NMR analysis of ionic liquids

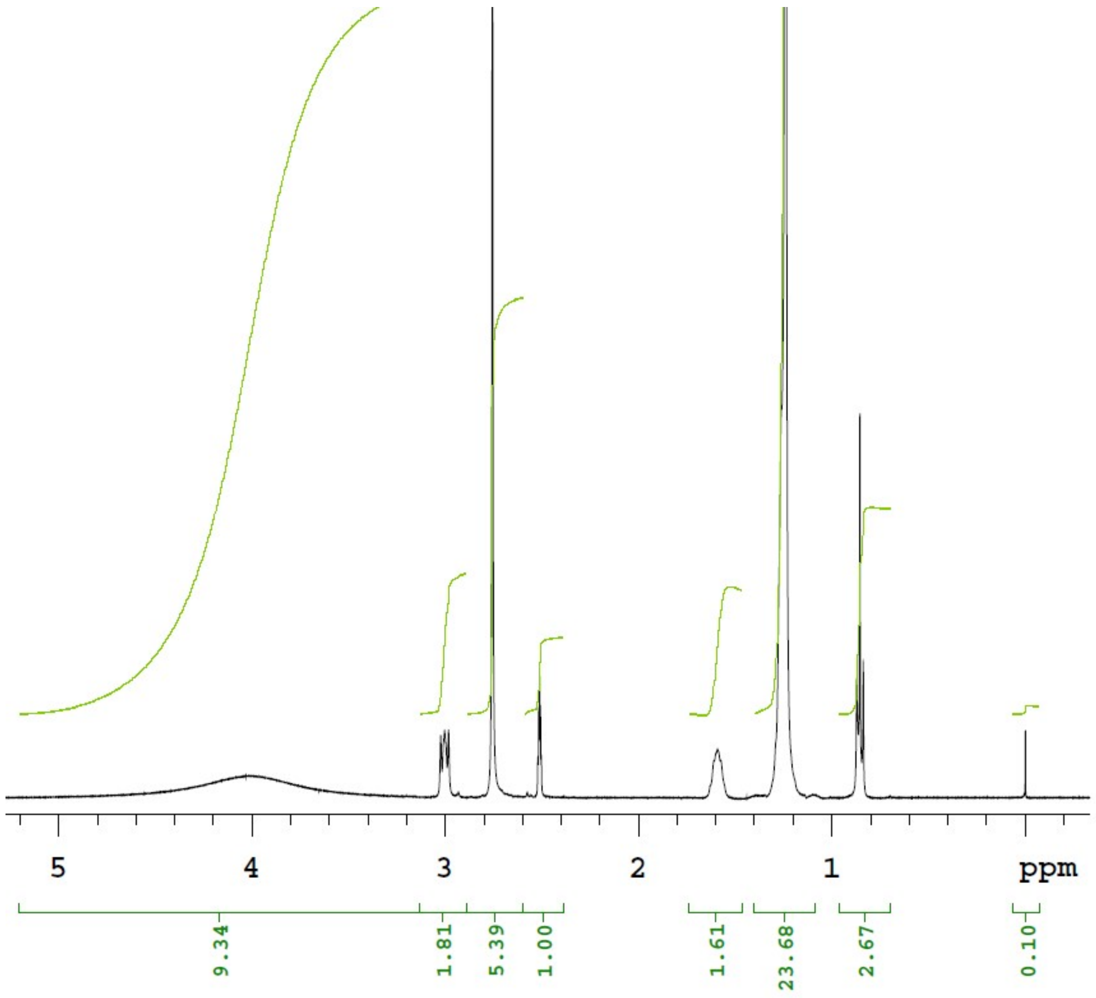

Fig. S1 ^{1}H NMR spectrum of di(hexadecyldimethylammonium) sulphate compound.

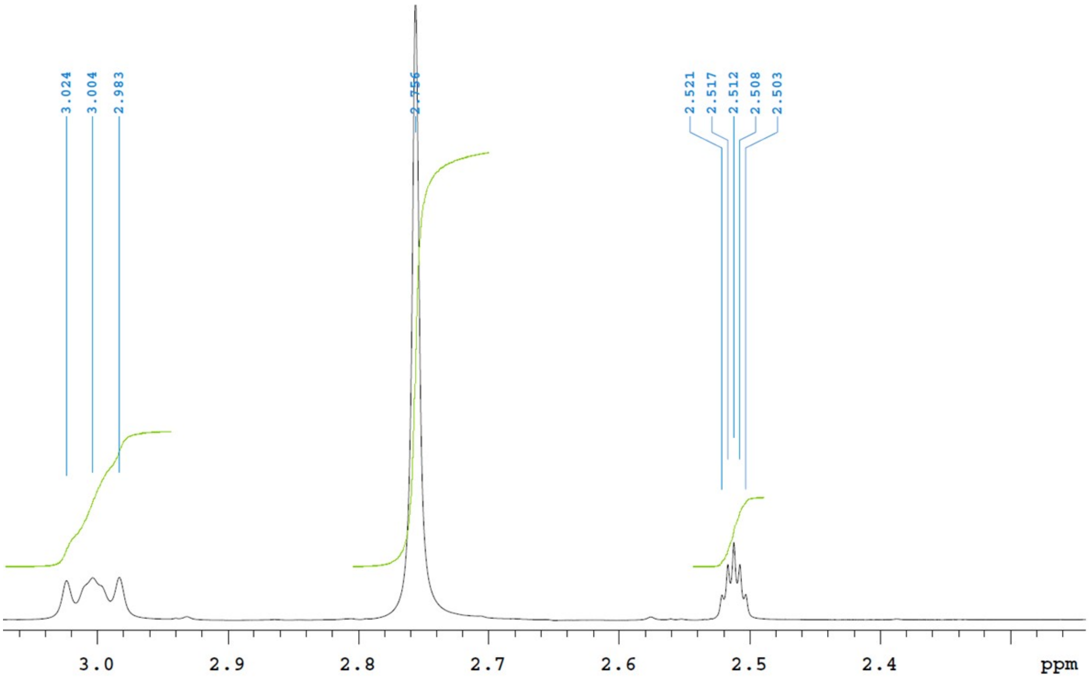

Fig. S2 ^{1}H NMR spectrum of di(hexadecyldimethylammonium) sulphate compound.

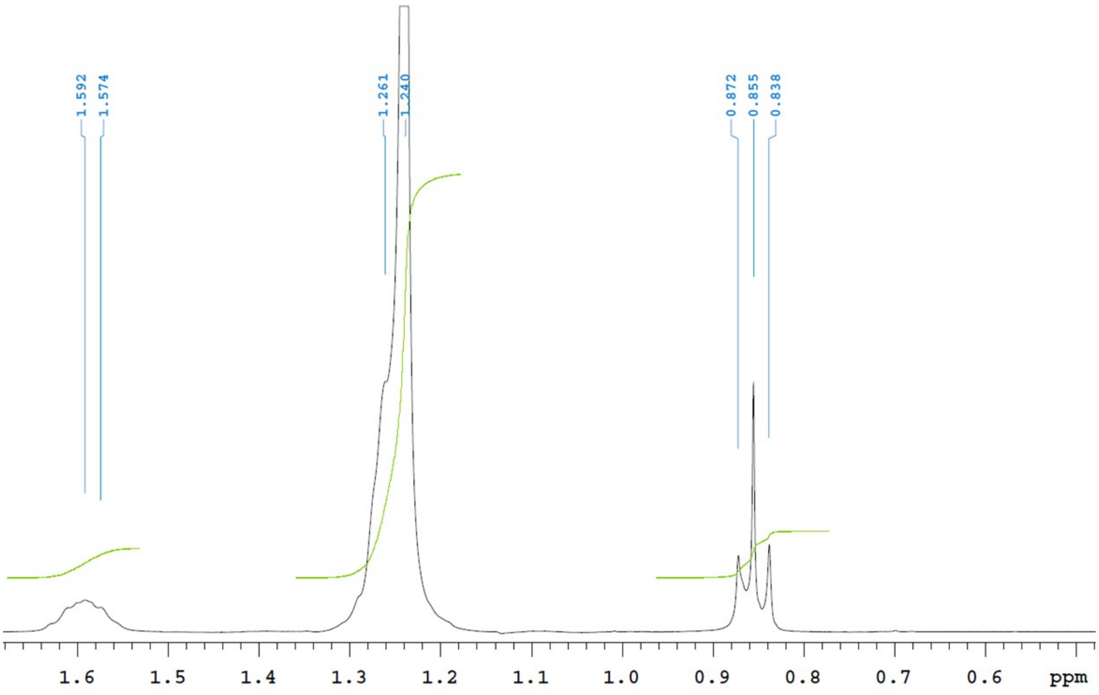

Fig. S3 ^{1}H NMR spectrum of di(hexadecyldimethylammonium) sulphate compound.

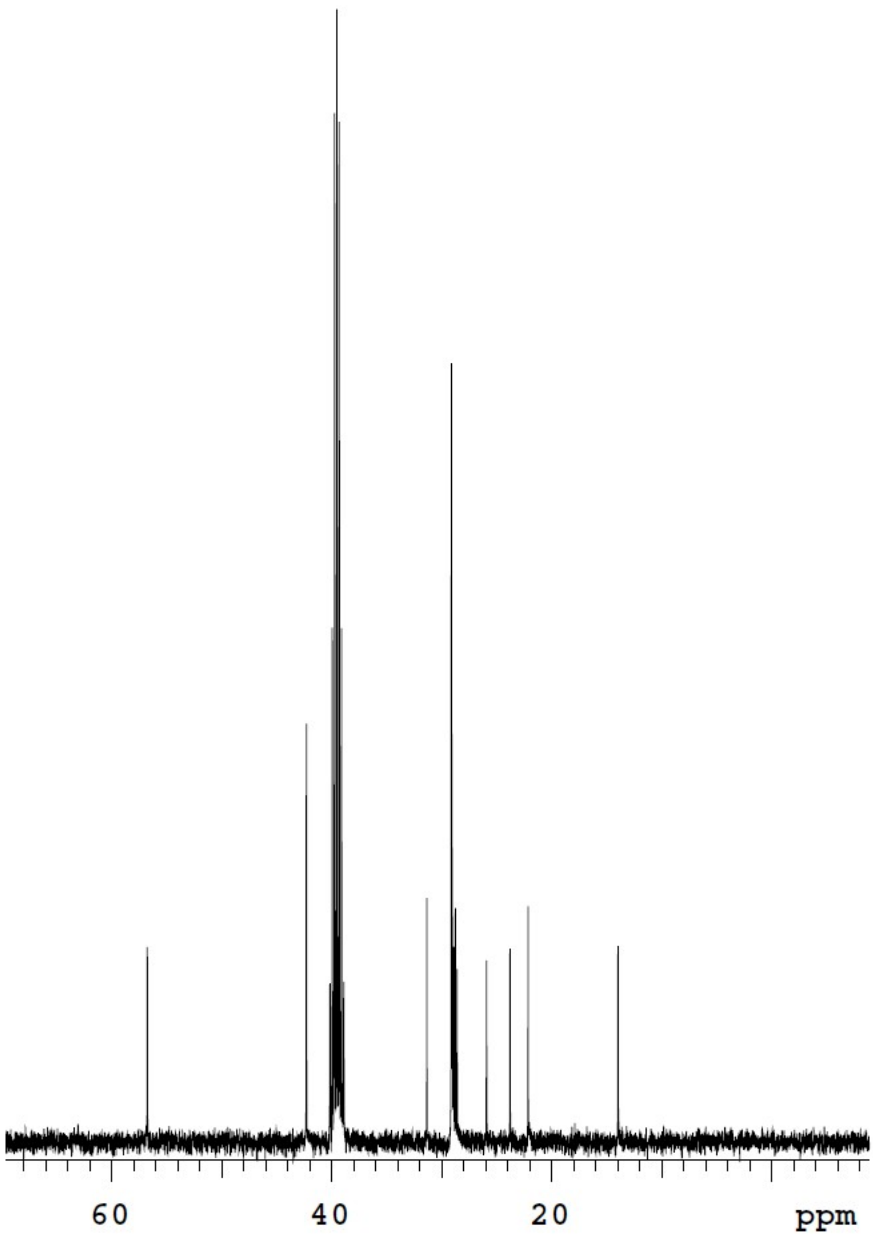

Fig. S4 ^{13}C NMR spectrum of di(hexadecyldimethylammonium) sulphate compound.

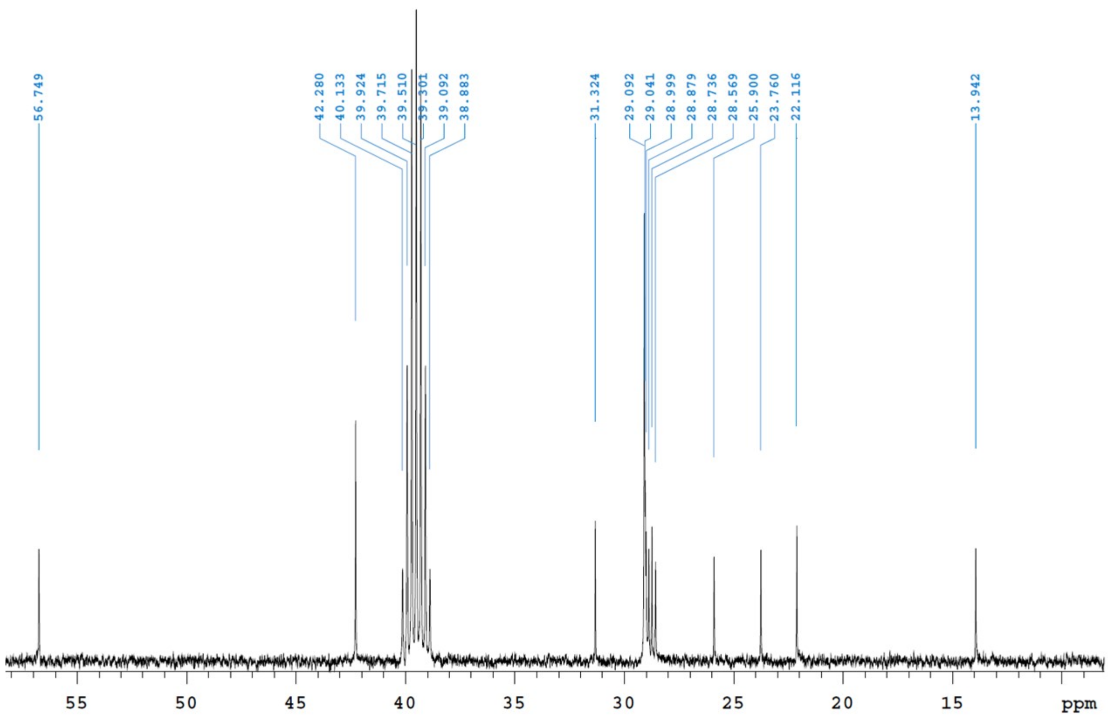

Fig. S5 ^{13}C NMR spectrum of di(hexadecyldimethylammonium) sulphate compound.

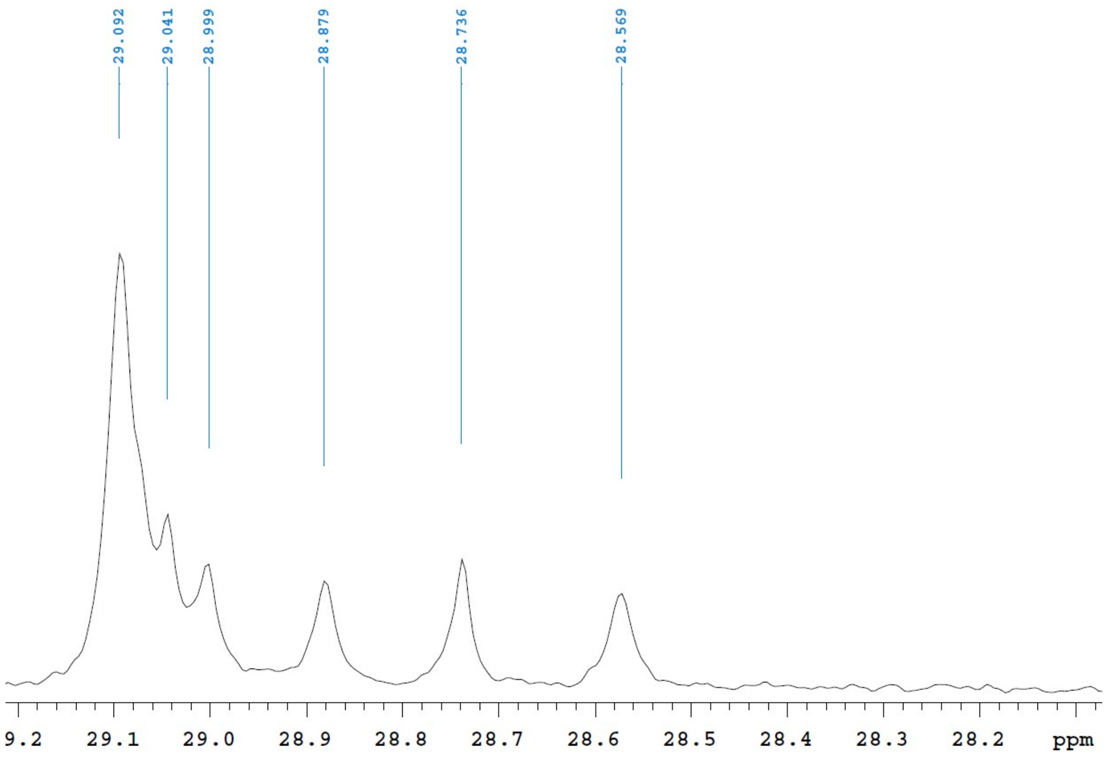

Fig. S6 ^{13}C NMR spectrum of di(hexadecyldimethylammonium) sulphate compound.

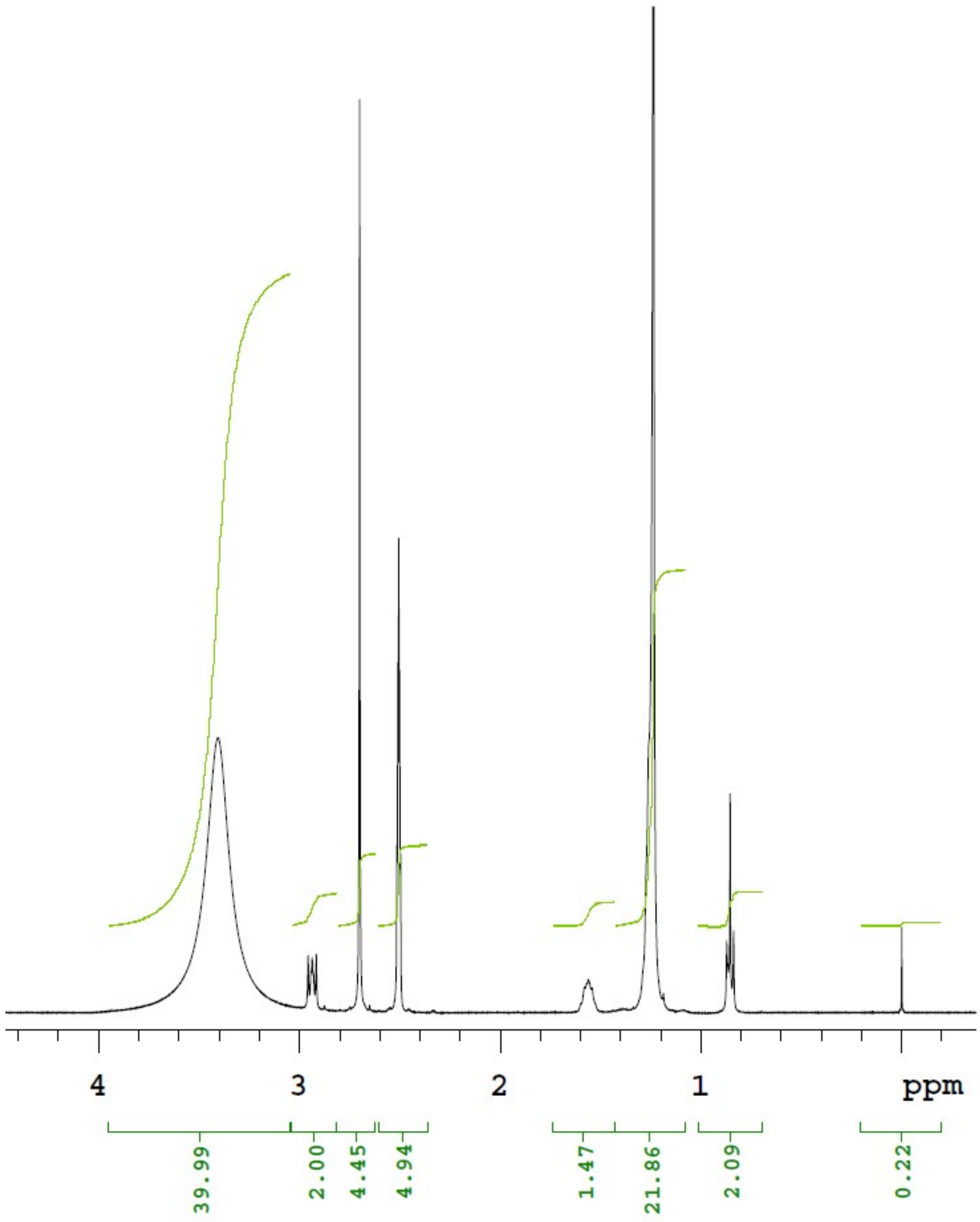

Fig. S7 ^{1}H NMR spectrum of di(octadecyldimethylammonium) sulphate compound.

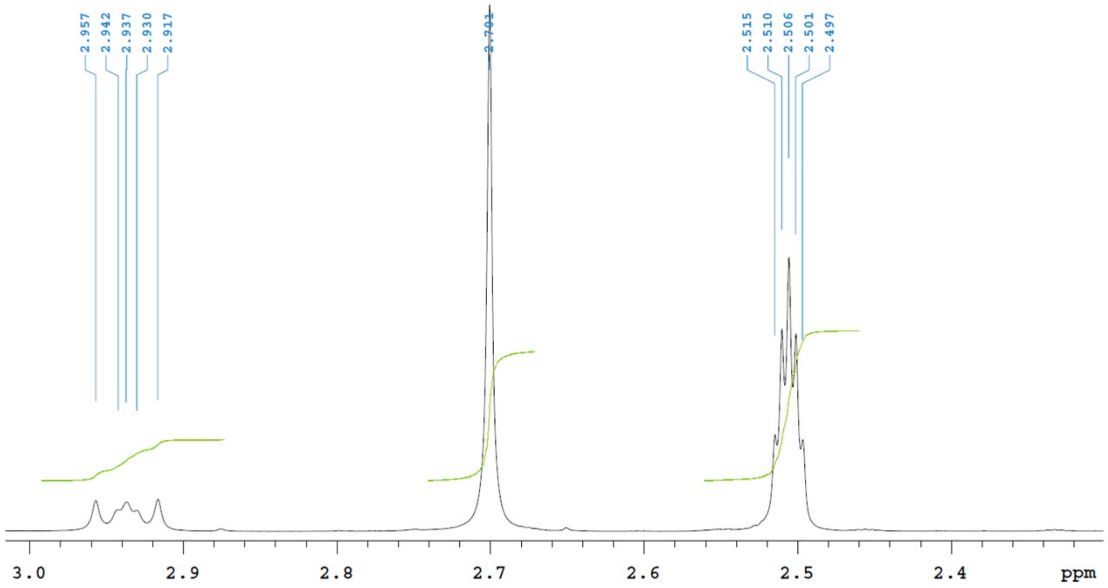

Fig. S8 ^{1}H NMR spectrum of di(octadecyldimethylammonium) sulphate compound.

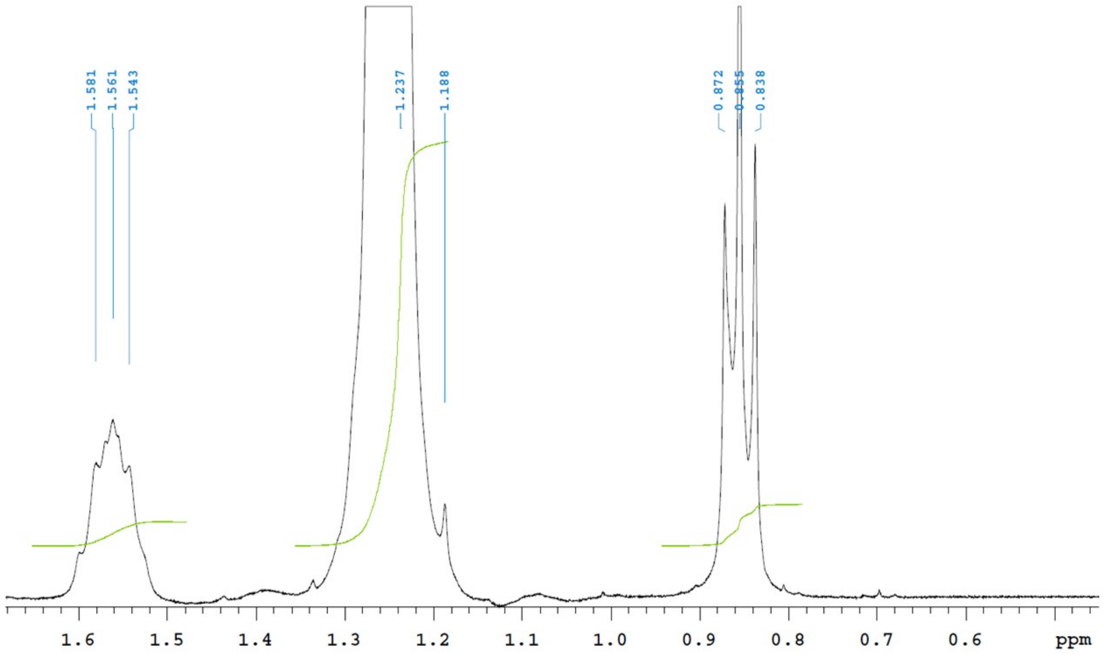

Fig. S9 ^{1}H NMR spectrum of di(octadecyldimethylammonium) sulphate compound.

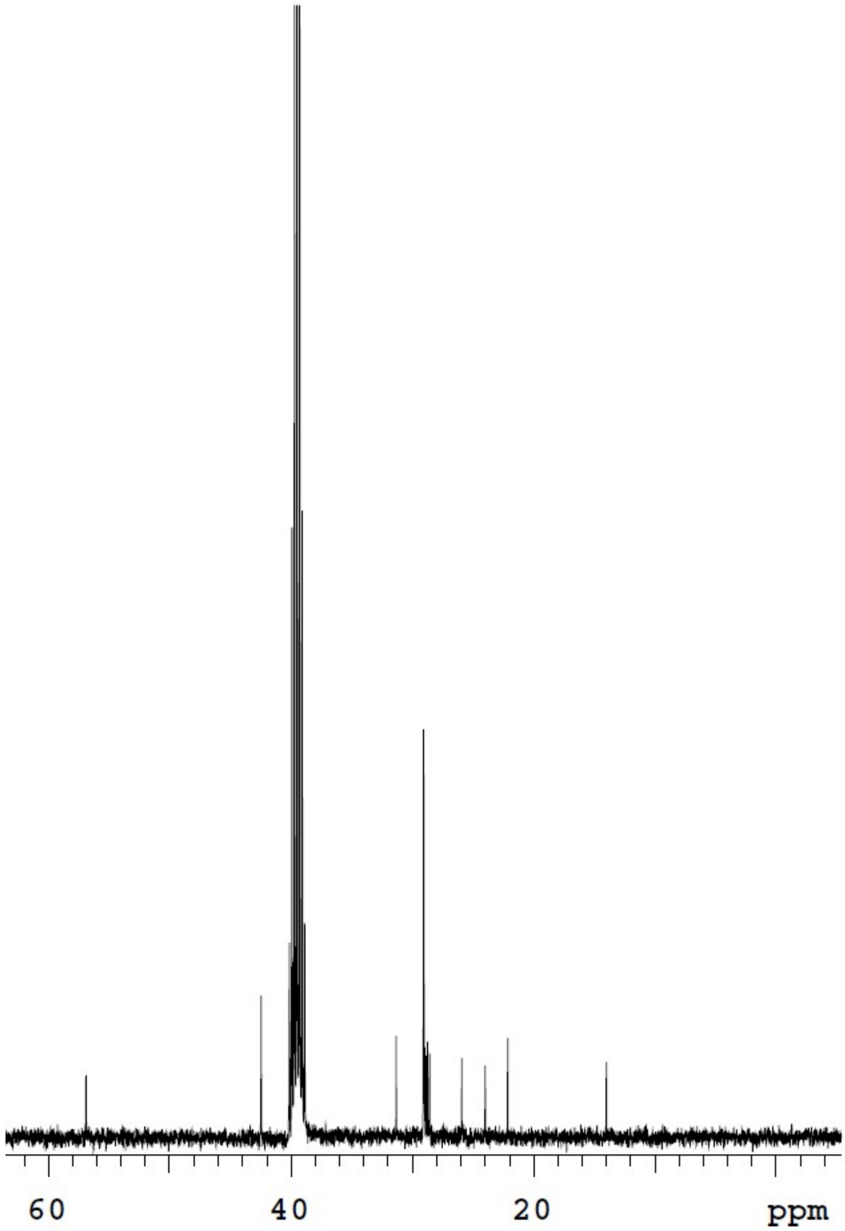

Fig. S10 ^{13}C NMR spectrum of di(octadecyldimethylammonium) sulphate compound.

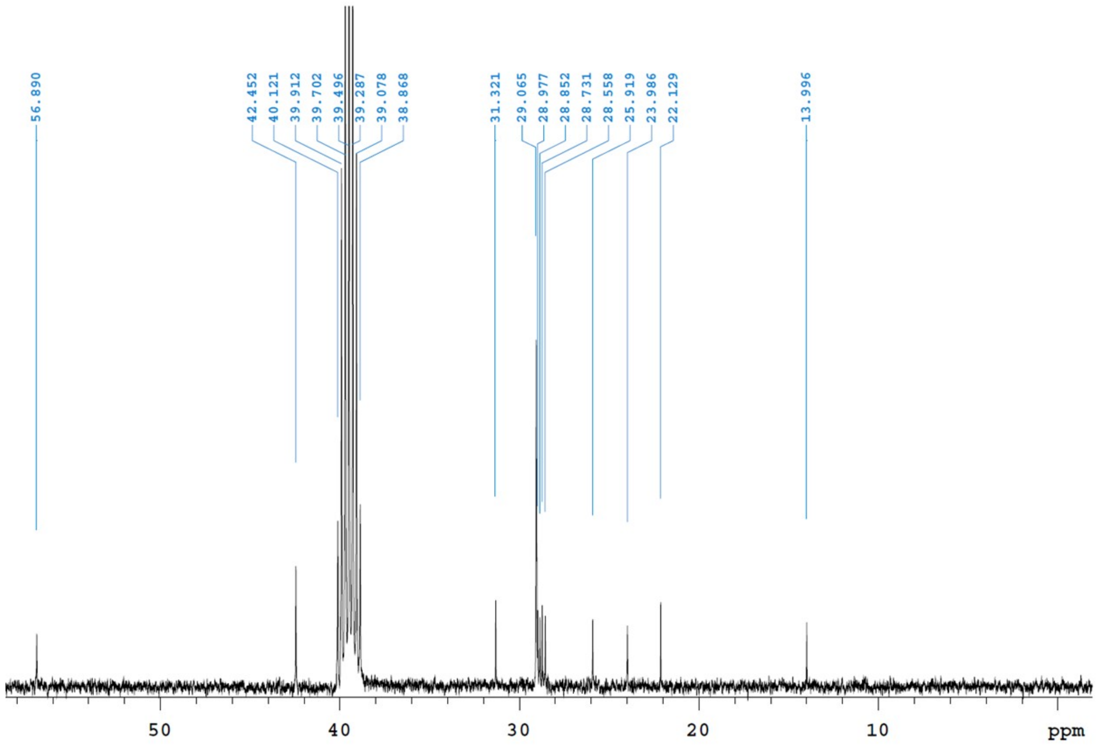

Fig. S11 ^{13}C NMR spectrum of di(octadecyldimethylammonium) sulphate compound.

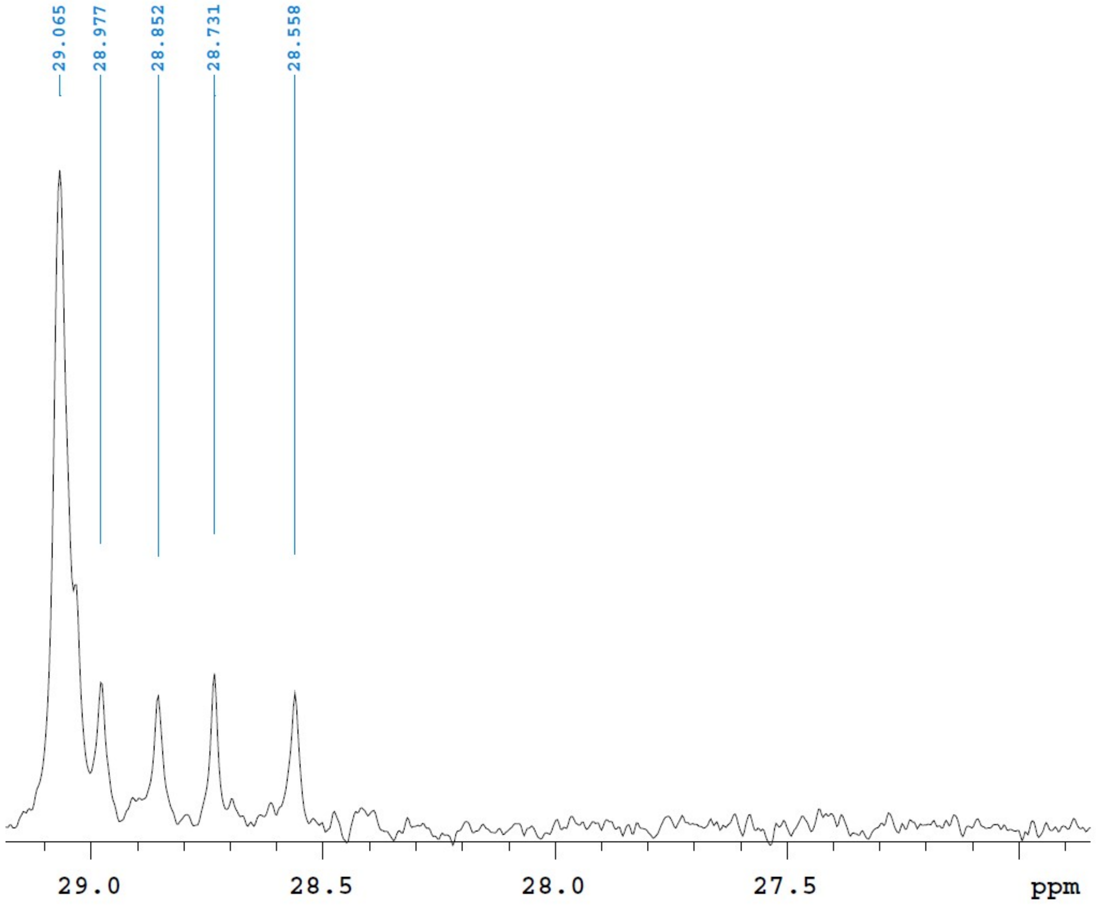

Fig. S12 ^{13}C NMR spectrum of di(octadecyldimethylammonium) sulphate compound.
